# A Previously Unknown Unique Challenge for Inhibitors of SYK ATP-Binding Site: Role of SYK as A Cell Cycle Checkpoint Regulator^☆^

**DOI:** 10.1016/j.ebiom.2014.10.019

**Published:** 2014-10-30

**Authors:** Fatih M. Uckun, Hong Ma, Zahide Ozer, Patricia Goodman, Jian Zhang, Sanjive Qazi

**Affiliations:** a Children's Center for Cancer and Blood Diseases, Children's Hospital Los Angeles, Los Angeles, CA 90027, USA; b Department of Pediatrics, University of Southern California Keck School of Medicine, Los Angeles, CA 90027, USA; c Molecular Oncology Program, Parker Hughes Institute, St. Paul, MN 55113, USA; d Department of Veterinary and Biomedical Science, University of Minnesota, St. Paul, MN 55108, USA; e Medicinal Bioinformatics Center, Shanghai Jiatong University, China; f Department of Biology and Bioinformatics Program, Gustavus Adolphus College, 800W College Avenue, St. Peter, MN 56082, USA

**Keywords:** Cell cycle, Tyrosine kinase, Phosphatase, Checkpoint control, Genomic instability

## Abstract

The identification of SYK as a molecular target in B-lineage leukemia/lymphoma cells prompted the development of SYK inhibitors as a new class of anti-cancer drug candidates. Here we report that induction of the SYK gene expression in human cells causes a significant down-regulation of evolutionarily conserved genes associated with mitosis and cell cycle progression providing unprecedented evidence that SYK is a master regulator of cell cycle regulatory checkpoint genes in human cells. We further show that SYK regulates the G_2_ checkpoint by physically associating with and inhibiting the dual-specificity phosphatase CDC25C via phosphorylation of its S216 residue. SYK depletion by RNA interference or treatment with the chemical SYK inhibitor prevented nocodazole-treated human cell lines from activating the G_2_ checkpoint via CDC25C S216-phosphorylation and resulted in polyploidy. Our study provides genetic and biochemical evidence that spleen tyrosine kinase (SYK) has a unique role in the activation of the G_2_ checkpoint in both non-lymphohematopoietic and B-lineage lymphoid cells. This previously unknown role of SYK as a cell cycle checkpoint regulator represents an unforeseen and significant challenge for inhibitors of SYK ATP binding site.

## Highlights

• SYK is a cell cycle regulatory kinase that phosphorylates CDC25C at S216
• SYK is a master regulator of cell cycle regulatory checkpoint genes in human cells
• Inhibitors of SYK ATP binding site may increase the risk for secondary cancer

## 1 Introduction

CDC25C is a dual specificity phosphatase that controls entry into mitosis (viz.: prophase to metaphase transition) by dephosphorylating p34^cdc2^/CDK1 on threonine 14 (T14) and tyrosine 15 (Y15) and thereby activating the CDK1/cylin B complex, also known as the mitosis promoting factor (MPF), at the end of G_2_ (Kiyokawa and Ray, 2008, Perry and Kornbluth, 2007, Donzelli and Draetta, 2003). S216 phosphorylation of CDC25C has been shown to inhibit its MPF-activating function in the nucleus by enhancing its binding to 14-3-3 proteins and thereby causing its sequestration in the cytoplasm (Kumagai and Dunphy, 1999). CDC25C is a key element of the G_2_ checkpoint pathway that delays entry into mitosis in response to DNA damage or microtubule-destabilizing agents such as nocodazole (NOC). In most species, the G_2_ checkpoint prevents CDC25C phosphatase from removing the T14/Y15 phosphate groups on CDK1 and thereby provides more time for DNA damage repair. This is accomplished by maintaining CDC25C in a phosphorylated form on its critical S216 residue in humans and the corresponding S287 residue in *Xenopus* (Kiyokawa and Ray, 2008). The checkpoint kinases, CHK1 and CHK2 are known to phosphorylate CDC25C on its S216 residue (Kiyokawa and Ray, 2008, Perry and Kornbluth, 2007). While some kinases, including PKA, C-TAK, and CAMKII have been shown to phosphorylate S287, they are not regulated by cell cycle checkpoints (Kiyokawa and Ray, 2008, Peng et al., 1998, Duckworth et al., 2002, Hutchins et al., 2003). It is generally assumed that additional G_2_ checkpoint kinases must exist but their identities have not yet been deciphered (Kiyokawa and Ray, 2008).

Spleen tyrosine kinase (SYK) is a physiologically important kinase that serves as a key regulator of multiple biochemical signal transduction events and biologic responses (Cheng et al., 1995, Mocsai et al., 2010, Turner et al., 1997, Uckun and Qazi, 2010, Zhou et al., 2006, Goodman et al., 2001, Heizmann and Reth, 2010, Wang et al., 2005, Uckun et al., 2010a, Uckun et al., 2010b, Uckun et al., 2012, He et al., 2002). We now provide new genetic and biochemical evidence that SYK is an inhibitor of CDC25C in B-lineage lymphoid cells as well as non-lymphohematopoietic cells, that prevents premature entry into mitosis by phosphorylating CDC25C at S216 when G_2_ checkpoint responses are activated.

## 2 Methods

### 2.1 Standard Biochemical, Imaging, and Transfection Methods

Confocal Laser Scanning Microscopy, co-immunoprecipitations, kinase assays, Western blot analyses, and gel filtration were performed as per previously described standard procedures (Uckun et al., 2010a, Uckun et al., 2010b, Uckun et al., 2012) (Supplemental information). 293T cells were transfected after reaching 70–80% confluence using ON-TARGET*plus* SMARTpool siRNA and DharmaFECT Transfection Reagent 4 (Catalog No. T-2004) (Thermo Scientific Dharmacon, Lafayette, CO, USA). The SYK phosphorylation site of CDC25C was determined by matrix-assisted laser desorption/ionization–time-of-flight (MALDI-TOF/TOF) mass spectrometry following a standard protocol (Supplemental information).

### 2.2 Molecular Model of SYK–CDC25C Interaction

A structural model of SYK–CDC25C peptide complex was constructed based on the ternary complex structure of PhK with MC peptide and then minimized using the Amber forcefield. While the structure of the C-terminal catalytic domain of CDC25C is known (PDB 3op3), the N-terminus, including the region corresponding to residues 211 to 219, does not have a known structure. Chen et al. built a kinase–substrate peptide model for the interaction of Chk1 with the human CDC25C peptide LYR**S**PSMPE (residue 211–219) based on the ternary complex structure of glycogen phosphorylase kinase (PhK) with a “Modified Cantley” (MC) peptide RQM**S**FRL (Chen et al., 2000). Using a modification of their reported strategy, we built a model for the binary SYK–CDC25C peptide complex. Specifically, we first superimposed the main-chain atoms of the crystal structure of the PhK–MC peptide complex (PDB entry: 2PHK) (Chen et al., 2000) and the *apo* SYK tyrosine kinase domain (PDB entry: 1XBA) (Atwell et al., 2004) using Sybyl6.8 (Tripos, St. Louis, MO). The MC peptide positioned in the superimposed SYK catalytic site, was then used as a template for “grafting” the 7-amino acid CDC25C peptide RSPSMPE, residues 213–219 (the underlined residue represents the predicted phosphorylation site) in backbone conformation into the SYK catalytic site according to the following sequence alignment, as previously reported by Chen et al. (2000).

$$\begin{array}{l} \mathrm{MC}\;\mathrm{Peptide}\;RQM\underline{S}FRL\\ \mathrm{CDC}25\;\mathrm{Peptide}\;RSP\underline{S}MPE \end{array}$$

An initial model of the SYK–CDC25C complex was obtained by randomly selecting the positions for CDC25C residues Tyr212 and Leu211 from the N-terminal of CDC25C Arg213 residue through residue conformational search encoded in the “Biopolymers” module of Sybyl6.8 and modeling the 9-amino acid CDC25C peptide (sequence LYRSPSMPE, residues 211–219) as a substrate in the SYK catalytic site. A two-step energy minimization of the CDC25C peptide within a radius of 6.5 Å around the catalytic site of SYK was carried out using Sybyl6.8 with the AMBER force field. The SYK–CDC25C peptide complex structure was minimized by first using the simplex method and then the Powell method to the energy gradient < 0.05 kcal/(mol·Å). The optimized parameters were set as follows: the distance-dependent function of the dielectric constant was adopted, non-bonded cutoff was set to 8 Å, and Amber charges were applied for the protein and peptide, as described by Zhang et al. (2005). The SYK–CDC25C peptide complex structure was minimized by first using the simplex method and then the Powell method to the energy gradient < 0.05 kcal/(mol·Å).

### 2.3 DNA Flow Cytometry

Cells (5 × 10^5^ per mL in plastic tissue culture flasks) were examined by DNA flow cytometry for emergence of polyploid cells after nocodazole exposure using standard procedures. Propidium iodide (PI, Sigma) was used to determine the percentages of cells in each phase of the cell cycle by quantitative DNA flow cytometry.

### 2.4 Establishment of U373 Cells with Ecdysone-Inducible SYK Gene Expression

U373 cells were transfected with the ecdysone inducible system regulatory vector, pVgRXR, and with a pIND/GS vector containing the cDNA encoding wildtype human *SYK* gene (H-L28824MI) (Invitrogen) using published procedures (Supplemental information).

## 3 Results

### 3.1 SYK is Localized to Centrosomes and Controls Expression Levels of G_2_ Checkpoint Genes in Human Cells

**Fig. 1:**
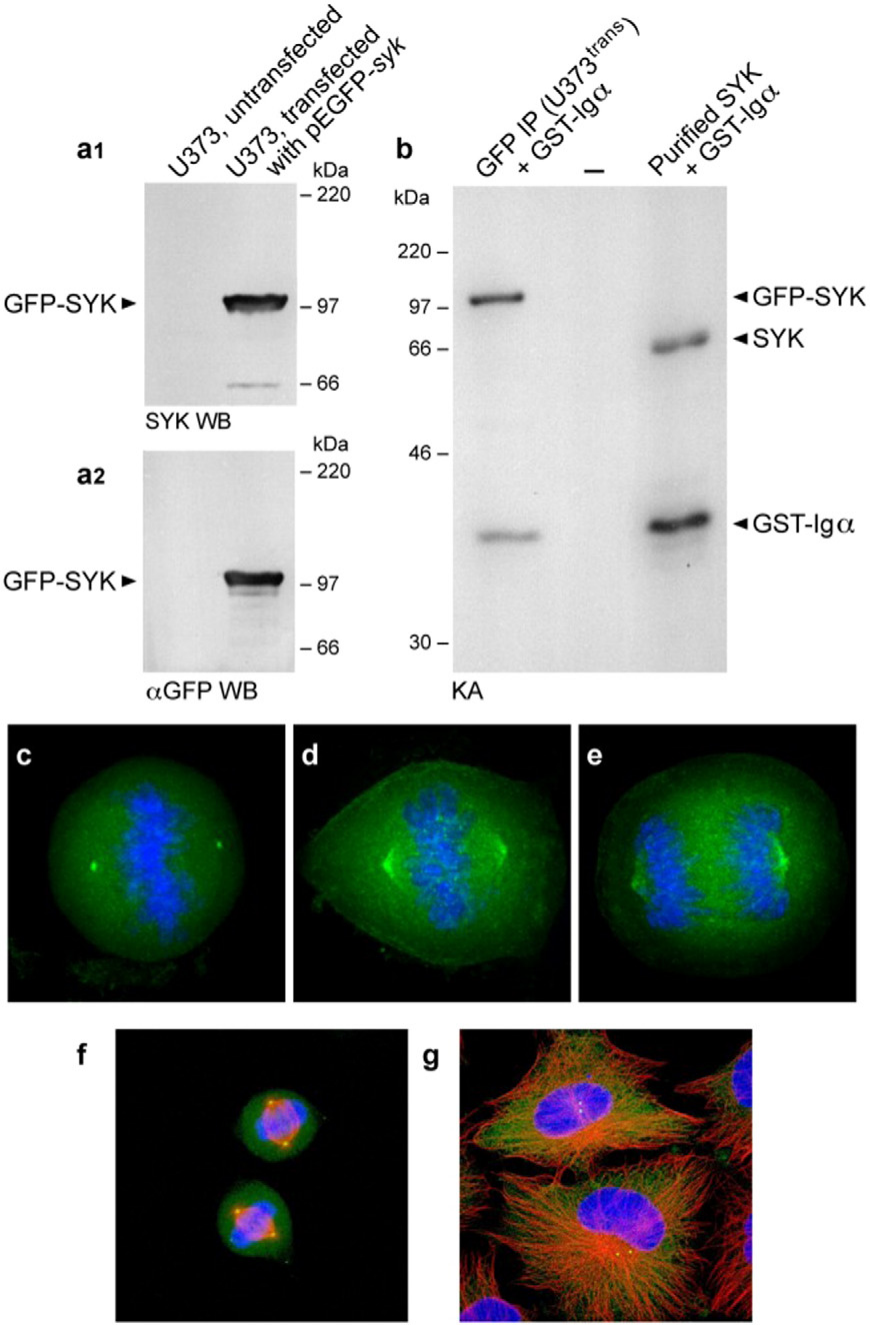
Subcellular localization of GFP-tagged recombinant rat SYK protein in transfected U373 human glioblastoma cells. [a.1 & a.2] Western blot analysis of whole cell lysates of U373 cells transfected with *pEGFP-SYK* plasmid (but not the whole cell lysates of untransfected U373 cells) with anti-SYK (Panel a.1) or anti-GFP (Panel a.2) antibodies confirmed abundant expression of a 99-kDa GFP-SYK fusion protein. [b] Immune complex kinase assays using monoclonal anti-GFP antibody for immunoprecipitation of GFP-tagged SYK and GST-Igα as an exogenous SYK kinase substrate confirmed the kinase activity of GFP-SYK expressed in U373 cells. Purified murine SYK protein together with GST-Igα as an exogenous SYK kinase substrate was included as a control. Two bands (marked with arrowheads) were identified in the kinase reaction mixture containing immunoprecipitates from *pEGFP-SYK* transfected cells. One band corresponds to autophosphorylation of GFP-tagged SYK and the other corresponds to phosphorylation of GST-Igα by GFP-tagged SYK. Likewise, purified recombinant murine SYK showed autophosphorylation and phosphorylated GST-Igα in the control kinase reaction. [c –e] When TOTO-3 stained transfected cells were analyzed by deconvolution microscopy, green-fluorescent GFP-tagged SYK localized as a single focus on each side of the metaphase plate to the mitotic spindle poles as well as spindle fibers in mitotic cells (d: metaphase, e: anaphase), easily identified by TOTO-3 stained (Blue) chromosomal DNA aligned on the metaphase plate (system magnification, 500×). [f & g] Transfected U373 cells were stained with TOTO-3 and a monoclonal anti-Tubulin antibody. Merge confocal images depicting centrosomal localization of GFP-tagged SYK are shown for interphase as well as metaphase cells. Panel f: Depicted are two representative metaphase cells. During metaphase, there is a single SYK focus localized to the centrosome at each spindle pole. Panel g: Depicted are two representative interphase cells. SYK staining in perinuclear centrioles is observed as two bright foci adjacent to the nucleus in interphase U373 cells. Green = GFP-tagged SYK; red = α-Tubulin; blue = TOTO-3 stained DNA/chromosomes (system magnification, 250 ×).

By using deconvolution microscopy and high-resolution confocal laser scanning microscopy, we first examined the subcellular localization of GFP-tagged recombinant SYK protein in the U373 human glioblastoma cell line that was stably transfected with the eukaryotic *SYK* expression vector pEGFP-*SYK* (Fig. 1). In mitotic U373 cells, a significant portion of the overexpressed green-fluorescent recombinant SYK protein was localized to the mitotic spindle poles on each side of the metaphase plate and spindle fibers consistent with a centrosomal localization (Fig. 1c–f). Likewise, SYK was detected in perinuclear centrioles of U373 cells in interphase (Fig. 1g).

**Fig. 2:**
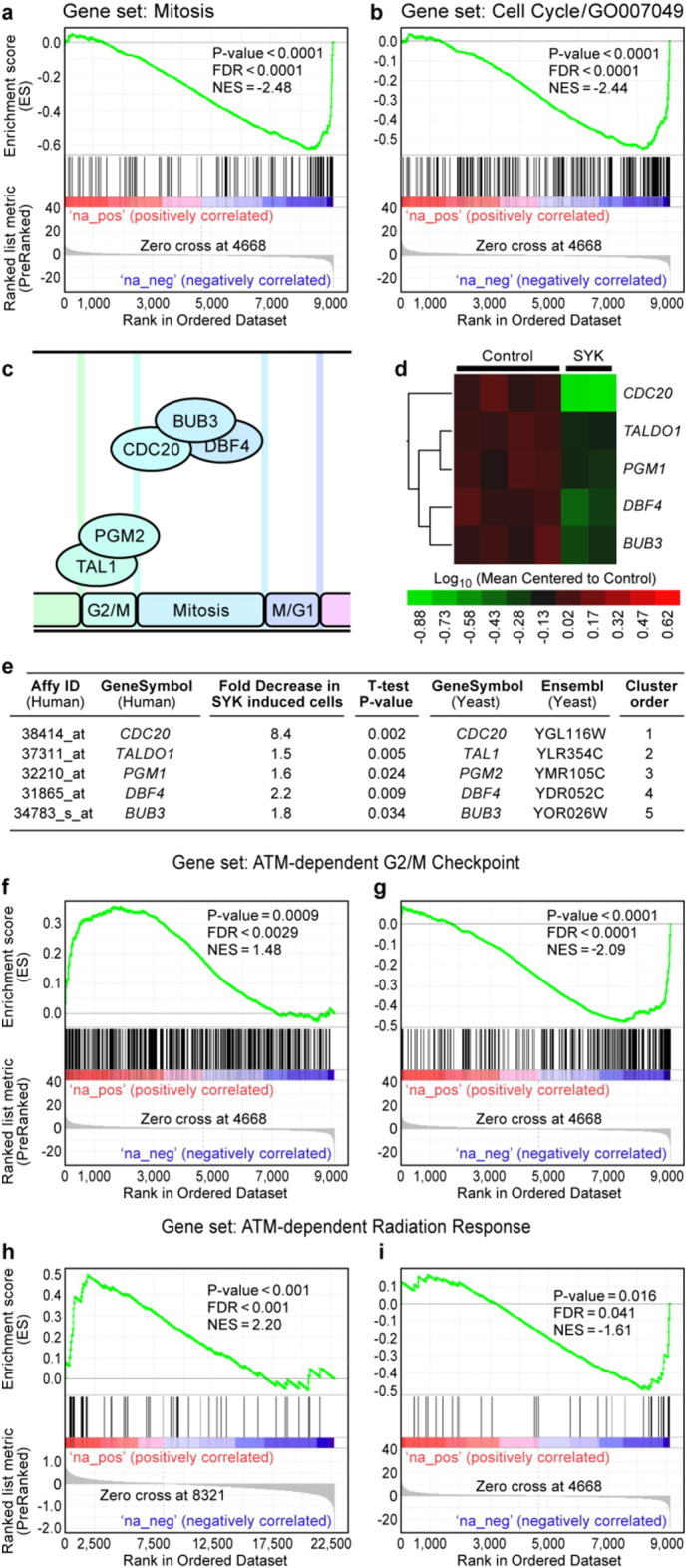
Induction of SYK gene expression causes down-regulation of cell cycle genes associated with G_2_/M transition. [a & b] Gene set enrichment analysis. Rank ordered T-values for SYK induced samples (red to blue up regulated to down regulated respectively) were processed for enrichment of Biological Process Gene Ontology terms using a supervised approach implemented in GSEA2.08 (Broad institute). Highly significant correlations (FDR < 0.0001) were observed for genes that were down regulated following SYK induction (hits shown by black lines on the graph) and annotations for “Mitotic Cell cycle” (a: Gene Set size = 112) and “Cell Cycle (GO 0007049)” (b: Gene Set size = 226). [c –e] SYK induction causes down-regulation of genes that exhibit peak expression in G_2_/M phases of the yeast cell cycle. The down-regulated genes in SYK-induced U373 cells included the human homologs of five yeast genes (viz.: *CDC20*, *TAL1*, *PGM2*, *DBF4*, *BUB3*) previously demonstrated to have peak expression in the G_2_ and M phases of the yeast cell cycle. Genome-wide gene expression data from highly synchronized yeast cultures were cross-referenced with human homologs of genes down-regulated by SYK induction in U373 cells (GSE18798). Five yeast genes exhibited primary peaks of expression at G_2_/M transition and in M-phase (c) depicted on the color-coded scaled diagram relating cell cycle phases and expression peaks (http://moment.utmb.edu/cgi-bin/timing_main.cgi). Human homologs of these yeast cell cycle genes were down-regulated in SYK-induced U373 cells (d). *CDC20* (8.4 fold decrease) was the most significantly down-regulated yeast G_2_/M gene (e). [f & g] SYK induction causes down-regulation of human homologs of ATM-dependent mouse G_2_/M checkpoint genes. Previously published global gene expression data (GSE11436) in thymocytes harvested from wildtype and ATM^−/−^ mice were compared to identify ionizing radiation (IR)-responsive genes that are ATM-dependent. Rank ordered T-values for SYK induced samples (red to blue up regulated to down-regulated respectively) were processed for enrichment of downstream targets of ATM in irradiated mice. f: The group of genes that were down-regulated in ATM^−/−^ cells was over-represented for mouse homologs of genes that were up-regulated with SYK induction (FDR = 0.0029; Gene set size = 388 genes). g: Conversely, the group of up-regulated genes in irradiated ATM^−/−^ cells was highly enriched for mouse homologs of genes that were down-regulated after SYK induction (FDR < 0.0001; Gene Set size = 235 genes). [h & i] SYK induction causes down-regulation of ATM-dependent human radiation response genes. Rank-ordered fold difference values from the comparison of irradiated wildtype and ATM^−/−^ mouse thymocytes (red indicates up-regulation and blue indicates down-regulation in ATM^−/−^ cells) and rank-ordered T-values for SYK-induced samples (red to blue: up-regulated to down-regulated) were processed for enrichment of downstream targets of human ATM. h: Radiation responsive human ATM targets (down-regulated in irradiated WT compared to irradiated ATM mutant human lymphoblasts) were overrepresented in genes up-regulated in irradiated thymocytes from ATM^−/−^ mice (P-value < 0.001, FDR < 0.001; probe set size = 52 Affymetrix Mouse 430A_2 gene chip probe sets representing 40 human orthologs). i: Radiation-responsive human ATM targets (down-regulated in irradiated WT compared to irradiated ATM mutant human lymphoblasts) were overrepresented in genes that were down-regulated after SYK induction (P-value = 0.016, FDR = 0.041; gene set size = 36 genes represented on the Affymetrix U95 Av2 genechip).

The centrosomal localization of SYK taken together with recent proteomic identification of several centrosomal proteins (Xue et al., 2012) as potential kinase substrates of SYK prompted the hypothesis that it may play an important role in cell cycle regulation. Pon-A Exposure of SYK-deficient U373 cells stably transfected with wildtype *SYK* gene induces expression of SYK and activates downstream signaling events mimicking oxidative stress-induced activation of SYK and SYK-dependent signal transduction pathways (Uckun et al., 2010a). In order to gain insights into the function of SYK as a centrosomal protein, we first examined the effect of SYK expression levels on the expression levels of cell cycle regulatory genes in human cells using this ecdysone-inducible mammalian expression system (Uckun et al., 2010a). The eukaryotic cell division cycle has been shown to rely on an intricate sequence of transcriptional events associated with distinct cell cycle regulated gene expression patterns (Rowicka et al., 2007). Gene set enrichment analysis (GSEA) showed that SYK induction in U373 cells causes a significant down-regulation of evolutionarily conserved genes associated with mitosis (Fig. 2a, normalized enrichment score: − 2.48, false discovery rate < 0.0001, P < 0.0001) and cell cycle progression (Fig. 2b, normalized enrichment score: − 2.44, false discovery rate < 0.0001, P < 0.0001). The down-regulated genes in SYK-induced U373 cells included the human homologs of five yeast genes (viz.: *CDC20*, *TAL1*, *PGM2*, *DBF4*, *BUB3*) (Fig. 2c–e) previously demonstrated to have peak expression in the G_2_ and M phases of the yeast cell cycle. Data for the cell cycle specific expression of these yeast genes was determined by high-resolution timing of cell cycle-regulated gene expression based on genome-wide gene expression data of synchronized yeast cultures (Rowicka et al., 2007). Among the 53 down-regulated genes, the most significantly affected 10 genes exhibiting the greatest fold-difference values were *PTTG1* (10.4-fold decrease, P = 0.0097), *UBEC2C* (8.5-fold decrease, P = 0.0033), *CDC20* (8.4-fold decrease, P = 0.002), *AURKA* (8.3-fold decrease, P = 0.0059), *CDC25C* (7.8-fold decrease ,GSE18798 P = 0.0076), *CCNB1* (7.4-fold decrease, P = 0.0045), *CCNB2* (6.8-fold decrease, P = 0.0029), *BUB1B* (6.4-fold decrease , P = 0.007), *BUB1* (5.6-fold decrease, P = 0.0047), and *SPAG5* (5.4-fold decrease, P = 0.0178) (accession #: GSE18798) (Fig. S1). In addition, 15 genes for key regulatory proteins with anti-proliferative functions such as *DUSP1* (3.7-fold increase, P = 0.0005), *SEPT4* (1.9-fold increase, P = 0.018), *SEPT7* (1.7-fold increase, P = 0.019), and *GAS1* (2.4-fold increase, P = 0.034) showed a moderate increase in expression after SYK induction (Fig. S1). The serine/threonine kinase ATM, encoded by the Ataxia telangiectasia-mutated (*ATM*) gene, is activated by DNA damage (viz.: double-stranded DNA breaks) and is required for G_2_ checkpoint activation, which is responsible for inhibition of G_2_/M transition following DNA damage (Innes et al., 2006, Stracker et al., 2008). In this context, ATM signaling delays the entry into mitosis by causing inactivation of CDC25C and thereby enforces the G_2_ checkpoint. ATM-dependent G_2_ checkpoint activation in irradiated mouse cells is associated with down-regulation of a unique group of highly correlated genes. Notably, the human homologs of many ATM-responsive G_2_ checkpoint signature genes were also down-regulated by induction of SYK expression in human U373 cells (Fig. 2f & g). A cluster of 2 genes (*AURKA and CCNB1*) showed greater than 5-fold decrease, a cluster of 3 genes (*CKS2*, *GAP43*, *NCAPD2*) showed greater than 3.5-fold decrease and a cluster of 3 genes (*HMGB2*, *FOXM1*, *NUDT1*) greater than 3-fold decrease in expression after SYK induction (Fig. S2). Likewise, a large panel of radiation-responsive genes that show down-regulation in ATM-deficient cells, exhibited increased expression levels in SYK-induced U373 cells, including *SERPINA1* that showed a 21.8-fold increase and a cluster of 12 genes (*COL4A5*, *CXADR*, *EFNB2*, *ETV5*, *GPC4*, *IGFBP5*, *IL1R1*, *P2RX4*, *PTPN13*, *PVRL3*, *RBMS1*, *VCAN*) with a greater than 2-fold increase (Fig. S3). Our GSEA also showed that human ATM targets (down-regulated in irradiated WT compared to irradiated ATM mutant human lymphoblasts) (Innes et al., 2006) were overrepresented in genes up-regulated in irradiated thymocytes from ATM^−/−^ mice (P-value < 0.001, FDR < 0.001) (Fig. 2h). Human ATM targets were overrepresented in genes that were down-regulated after SYK induction (P-value = 0.016, FDR = 0.041) providing evidence that SYK induction in U373 cells causes down-regulation of ATM-dependent human radiation response genes (Fig. 2I). This immediate response to SYK induction at the transcriptome level with altered expression of multiple evolutionarily conserved cell cycle genes served as a strong and compelling early indicator that SYK has a critical and previously unrecognized role in mitotic cell cycle regulation. The down-regulation of the human orthologs of yeast G_2_/M genes and human orthologs of ATM-dependent murine G_2_-checkpoint genes as well as ATM-dependent human radiation-response genes prompted the hypothesis that SYK induction may activate a G_2_ checkpoint GSE18798 (Accession #: GSE18798).

### 3.2 Role of SYK as a Kinase that Controls the Cell Cycle in Response to Microtubule and DNA damage

**Fig. 3:**
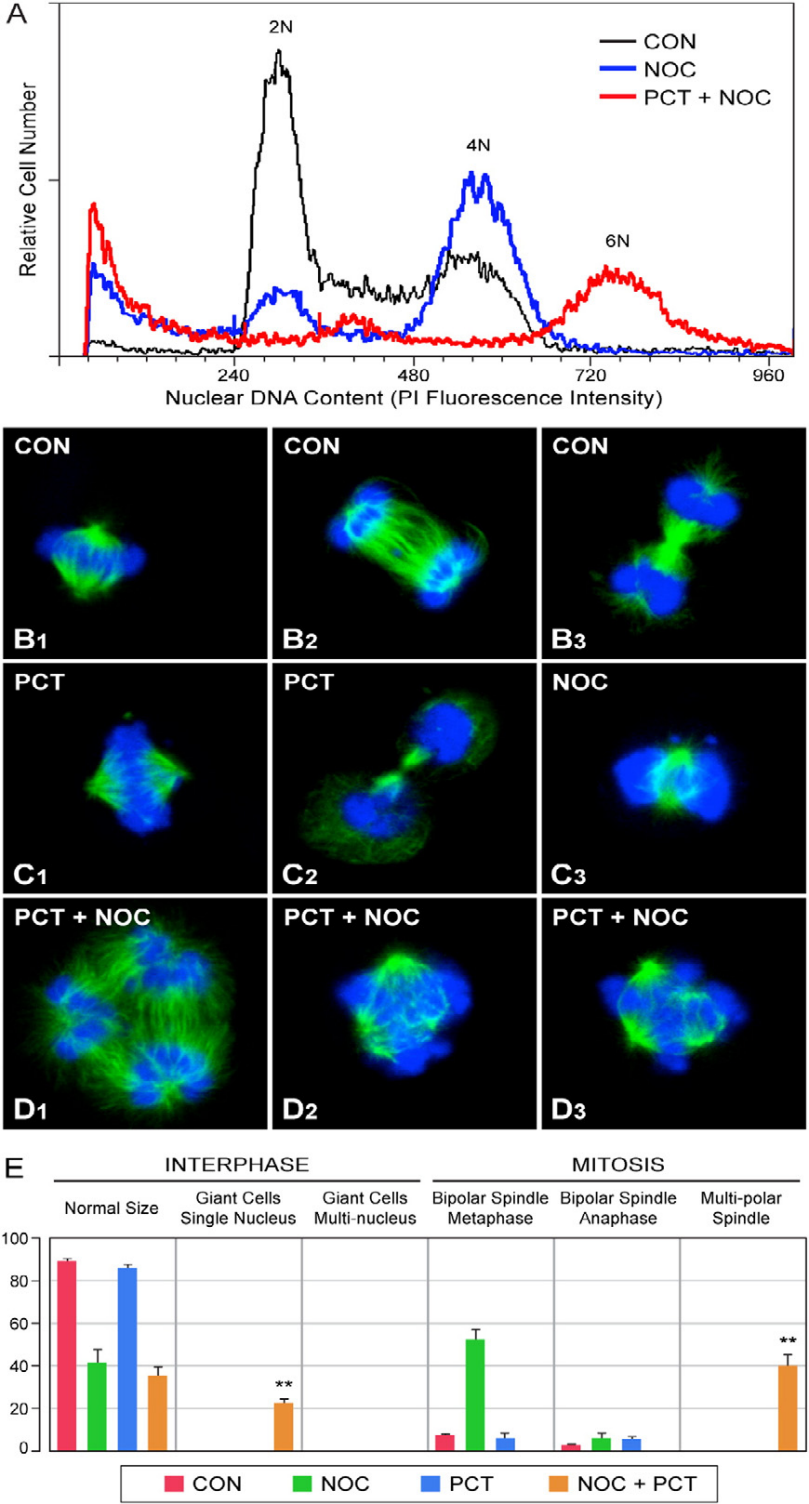
Effects of the SYK inhibitor PCT on nocadozole response of a human B-cell line. [a] Asynchronously growing EBV-transformed human lymphoblastoid B-cell line BCL1 was exposed to 0.03 μg/mL (100 nM) NOC for 48 h and then examined by DNA flow cytometry. [b–d] Depicted are representative two-color Tubulin (green)/DAPI (blue) confocal images of mitotic BCL-1 cells after 48 h treatment of BCL-1 cells with medium alone (b1–b3), medium supplemented with 30 μM PCT (c1, c2), medium supplemented with 100 nM NOC (c3), or medium supplemented with 30 μM PCT + 100 nM NOC (d1–d3). System magnification: 250×. b1, c1: Normal metaphase with a bipolar mitotic spindle. b2: Early telophase. b3: Late telophase with midzone microtubules. c2: Cytokinesis. c3. Abnormal metaphase as commonly observed in NOC-induced metaphase arrest  with lagging chromosomes and absence of correctly aligned chromosomes at the metaphase plate 52 ± 5% of BCL-1 cells treated with NOC were in metaphase arrest. In each of 3 independent experiments, NOC-treated BCL-1 cultures showed a significantly higher percentage of cells in metaphase than untreated control cells (Dunnett's test, P < 0.001) and exhibited chromosome alignment aberrations commonly seen in NOC-induced mitotic arrest. d1–d3: Multipolar spindles. 40 ± 5% of BCL-1 cells treated with PCT + NOC showed multipolar spindles. No normal metaphases or anaphases with bipolar spindles were observed. Multipolar spindles were observed only when BCL-1 cells were treated with NOC in the presence of PCT. e. Similar results were obtained in 3 independent experiments and the quantitative data for the % of cells displaying the noted characteristics are depicted in bar graphs. The average cell numbers analyzed microscopically for each treatment condition were 57 ± 10 cells in control cultures treated with medium alone, 41 ± 8 cells for NOC treatment; 57 ± 5 cells for PCT treatment and 47 ± 5 cells for NOC + PCT treatment. Columns showing the results for interphase cells: Mean ± SE values for percentages of interphase cells with a normal size single nucleus, “giant” polyploid interphase cells with a large single nucleus, and multinucleated interphase cells. Columns showing the results for mitotic cells: Mean ± SE values for percentages of metaphase cells with a bipolar spindle, anaphase cells with a bipolar spindle, and metaphase/anaphase cells with multipolar spindles. NOC-treated cultures showed a significantly higher percentage of cells in metaphase (Dunnett's test, P < 0.001) with chromosome alignment aberrations commonly seen in NOC-induced mitotic arrest. No multinuclear cells were observed in any of the cultures. Giant polyploid interphase cells and mitotic cells with abnormal multipolar spindles were observed only in cells treated with NOC + PCT (**, *P* < 0.001; Dunnett's test P-value vs CON).

**Fig. 4:**
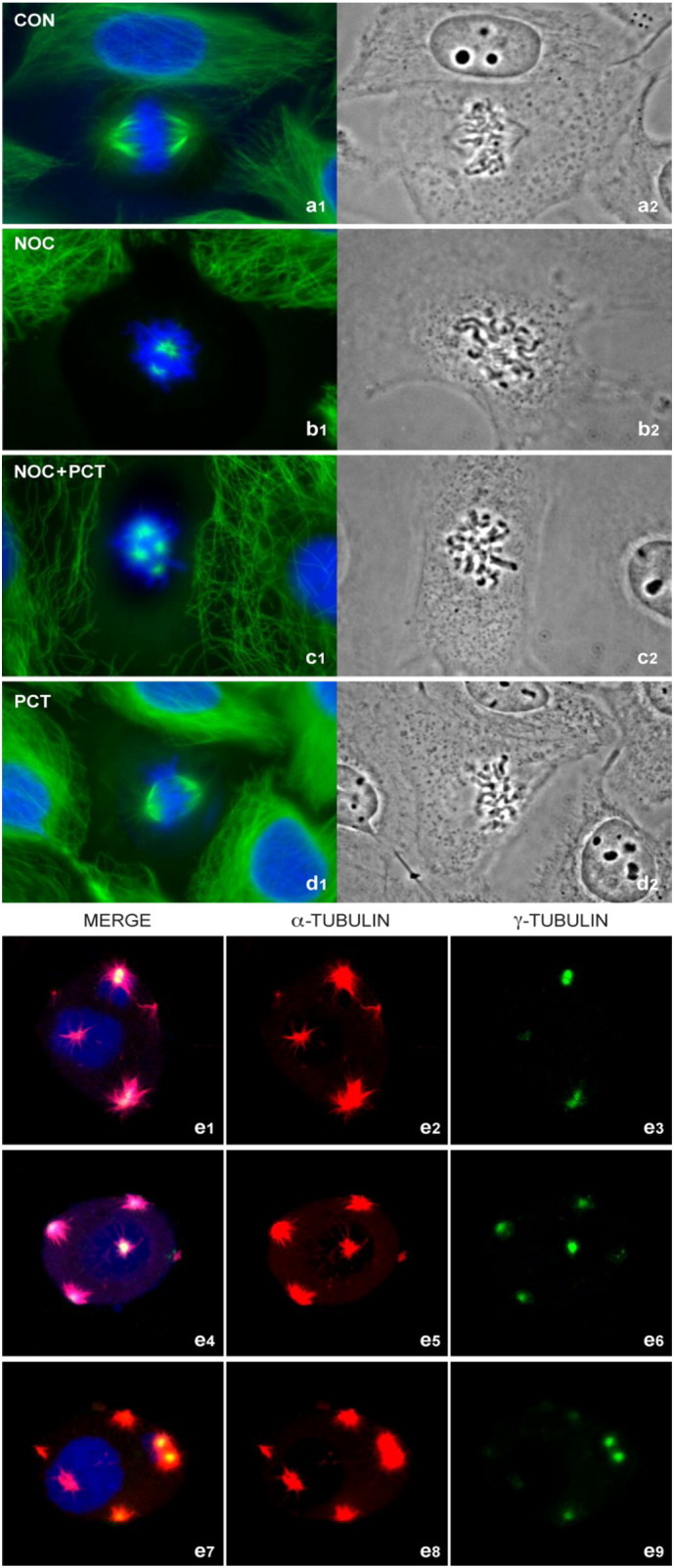
Effects of the SYK inhibitor PCT on NOC responses of BT20 human breast cancer cells. Fluorescence (a1–d1) and phase-contrast microscopy (a2–d2) images of BT20 cells in mitosis. System magnification: 250×. Panels e1 –e9 depict confocal images of three representative cells from NOC + PCT treated cultures stained with α-Tubulin (red), γ-tubulin (green), and DNA (DAPI, blue). γ-tubulin staining served as a centrosome marker. System magnification: 500×. While normal bipolar spindles were observed in untreated control or PCT-treated BT20 cells (a), NOC-treated BT20 cells showed abnormal metaphases with unaligned chromosomes congressed at a non-coherent metaphase plate (b). BT20 cells treated with NOC + PCT showed highly aberrant accumulation of condensed unaligned chromosomes in midzone (c) as well as multipolar spindles (e). Similar results were obtained in 3 independent experiments and the quantitative data are shown in Fig. S7, Panel b.

Treatment of mammalian cells with the microtubule-destabilizing agent nocodazole (NOC) causes mitotic arrest in the M-phase. When asynchronously growing EBV-transformed human lymphoblastoid B-cell line BCL1 was exposed to 0.03 μg/mL (100 nM) NOC for 48 h, the majority of the cells accumulated with a 4N DNA content, as determined by DNA flow cytometry (Fig. 3). However, in the presence of the SYK inhibitor piceattanol (PCT) (30 μM), NOC was unable to effectively cause an M-phase arrest in BCL1 cells and the majority of these cells accumulated with a > 4N DNA content (Fig. 3a). Confocal immunofluorescence microscopy of 48 h cultures of BCL-1 cells treated with NOC + PCT showed both mitotic cells with highly aberrant multipolar spindle formation (Fig. 3d1–d3). Examination of BT20 human breast cancer cells (Fig. 4) treated with NOC vs. NOC + PCT by fluorescence and phase-contrast microscopy yielded similar results. The failure of NOC to cause metaphase arrest in the presence of a SYK inhibitor uniquely indicated that SYK may control the cell cycle response to microtubule damage.

**Fig. 5:**
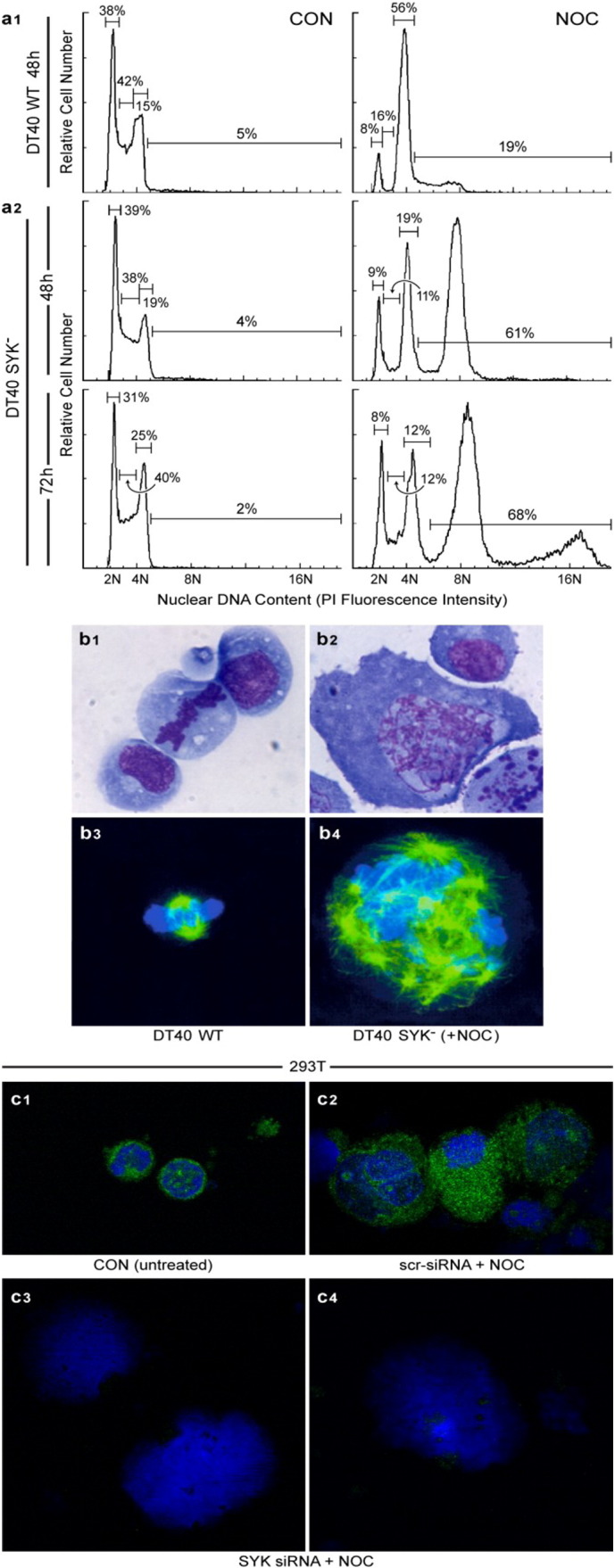
SYK gene is required for nocodazole-induced mitotic arrest. [a &b] DT40 chicken lymphoma B-cells were treated with NOC (0.12 μg/mL × 48 h at 37 °C) and then examined by DNA flow cytometry for emergence of polyploid cells. The decimal points for the percentages of nuclei with defined DNA content were rounded off in the depicted DNA histograms. [a.1] Wildtype DT40 cells that showed accumulation in G_2_/M after NOC treatment. The percentages of 2N, 4N and > 4N nuclei were 8.1%, 56.2%, and 19.3%, respectively and % of cells in S-phase was 16.3%. [a.2] A substantial proportion of SYK-deficient DT40 cells which were established by homologous recombination knockout, showed only a partial accumulation of cells with a 4N DNA content when treated with nocodazole, and > 50% of these cells continued their DNA synthesis beyond 4N nuclear DNA content. The aberrant DNA synthesis continued after cells were washed to remove NOC at 48 h with 68% of the cells showing 8N–16N DNA content at 72 h. At 72 h, 1.7% of untreated SYK-deficient DT40 cells had hypodiploid/apoptotic nuclei that are not included in the DNA histogram. [b.1 & b.2] Morphologic features of Nocodazole-treated SYK-Deficient DT40 cells. Wright–Giemsa stained cytospin slides of NOC-treated wildtype (b1) and SYK-deficient (b.2) cells were examined by light microscopy at 48 h post NOC exposure. More than 50% of NOC-treated SYK-deficient DT40 cells (but not wildtype DT40 cells) were very large mononuclear cells with partially decondensed chromosomes. System magnification: 100×. [b3 & b4] Confocal two-color fluorescence merge image of a representative untreated wildtype DT40 cell in metaphase with a bipolar mitotic spindle (b3) vs. a representative NOC-treated polyploid SYK-deficient (b4) DT40 cell with abnormal multipolar spindles. The images were obtained following a 48 h treatment with 0.12 μg/ml NOC. Green = Tubulin; blue = TOTO-3 stained DNA/Chromosomes (system magnification: 500 ×). [c] siRNA-induced depletion of native SYK causes polyploidy in Nocodazole-treated 293T cells. Confocal images of 293T cells stained with the fluorescent DNA dye 4 ′,6-diamidino-2-phenylindole (DAPI) (blue) and anti-SYK antibody (green) after 72 h of RNAi via transfection with SYK-siRNA or scrambled(scr)-siRNA (included as a control) and 48 h of treatment with 400 nM NOC (i.e. 120 h after the start of the RNAi.). Each siRNA was used at a 50 nM concentration. A no treatment control (CON) was also included. Twelve of 12 control 293T cells showed abundant SYK staining and normal size nuclei (c1). Six of 12 scr-siRNA transfected, NOC-treated 293T cells showed enlarged nuclei and abundant SYK expression (c2). The remainder of the scr-siRNA transfected, NOC-treated 293T cells had a normal size nucleus. Eight of 20 SYK-siRNA transfected 293T cells became polyploid after NOC exposure with very large nuclei and profoundly diminished SYK expression due to siRNA-mediated SYK depletion (c3 & c4). The remainder of the SYK-siRNA treated cells had an enlarged nucleus and profoundly diminished SYK expression due to siRNA-mediated SYK depletion (system magnification: 600×).

We next sought direct and unequivocal genetic evidence for a cell cycle regulatory role of SYK in lymphoid cells using DT40 chicken B-cell line and its SYK-deficient DT40 chicken B-cell lymphoma clones that were established by homologous recombination knockout (Uckun et al., 1996, Uckun et al., 2010a). When asynchronously growing wildtype DT40 cells were exposed to 0.12 μg/mL (400 nM) NOC for 48 h, 56% accumulated with a 4N DNA content and only 19% became polyploid, as determined by DNA flow cytometry (Fig. 5a1). In contrast to wildtype DT40 cells, only 19% of NOC-treated SYK-deficient DT40 cells had a 4N DNA content and 61% of these cells continued their DNA synthesis beyond 4N nuclear DNA content with emergence of 8N nuclei at 48 h and emergence of 8N and 16N nuclei at 72 h (Fig. 5a2). Light microscopic examination of Wright–Giemsa stained cytospin slides of NOC-treated wildtype vs. and SYK-deficient DT40 cells showed that more than 50% of NOC-treated SYK-deficient DT40 cells (but not wildtype DT40 cells) were very large mononuclear cells with partially decondensed chromosomes (Fig. 5, b1 vs. b2). High-resolution confocal microscopy of NOC-treated cultures of SYK-deficient DT40 cells showed very large mitotic cells with highly aberrant multipolar spindle formation (Fig. 5, b3 vs. b4). To further document the significance of SYK in cell cycle response to microtubule damage, we next examined the effects of SYK depletion by RNAi on NOC response of human 293T cells. SYK-siRNA causes selective depletion of SYK protein in 293T cells within 72 h, as confirmed by Western blot analysis (Uckun et al., 2010a, Uckun et al., 2012) and confocal microscopy (Fig. 5c). Notably, treatment with 50 nM SYK siRNA (but not scrambled control siRNA) for 72 h prevented NOC from causing a metaphase arrest and resulted in polyploidy, as determined by confocal microscopic examination of the size and DNA content of DAPI-stained nuclei (Fig. 5c). The striking SYK-dependency of the NOC response in these RNAi experiments further confirmed that SYK plays a critical role in the regulation of the cell cycle response to microtubule damage.

### 3.3 In Situ Physical Interactions Between Native SYK and CDC25C in Human Cells

**Fig. 6:**
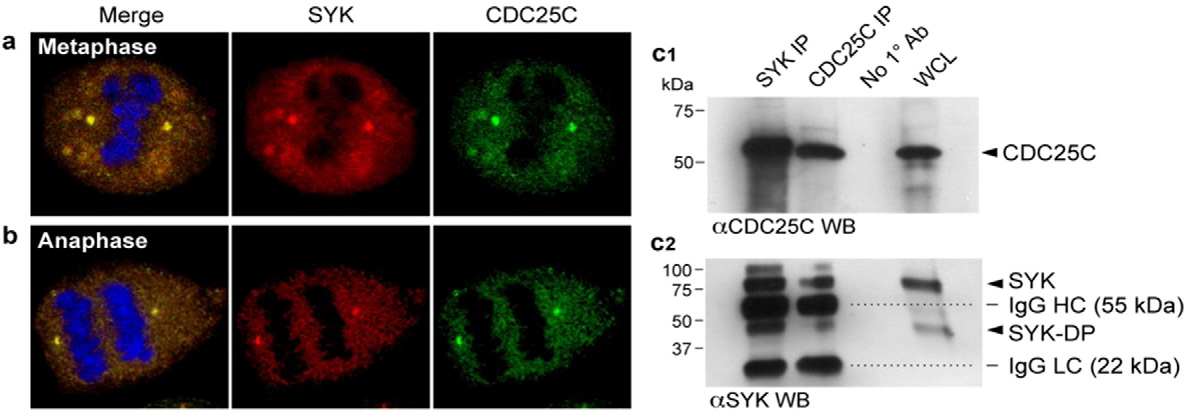
Centrosomal co-localization and physical interactions of native SYK and CDC25C proteins in human cells. [a & b] Subcellular co-localization of SYK and CDC25C during mitosis. Log-phase BT20 cells growing in culture were fixed and stained with either anti-SYK (red) or anti-CDC25C (green) antibodies. Cellular DNA was labeled in all slides with TOTO-3 (Blue). Depicted are representative cells in metaphase (a) or anaphase (b) showing subcellular localization of SYK and CDC25C. Merge confocal images document co-localization of SYK and CDC25C as “yellow” immunofluorescent foci. Chromosomes at the metaphase plate show blue fluorescence because of TOTO-3 labeled DNA. System magnification: 500 ×. [c] In Situ Association of SYK and *CDC25C*. [c1] CDC25C Western blot analysis of SYK and CDC25C immune complexes. SYK (lane 1) and CDC25C (lane 2) immune complexes immunoprecipitated from human LOUCY cell line were subjected to CDC25C Western blot analysis. [c2] SYK Western blot analysis of SYK and CDC25C immune complexes. The same SYK and CDC25C immune complexes immunoprecipitated from LOUCY cells that were examined by CDC25C Western blot analysis as depicted in Panel c1 were subjected to SYK Western blot analysis. The positions of CDC25C (predicted size: 53 kDa), SYK (predicted size: 72 kDa), a degradation product of native SYK (SYK-DP) as well as the positions for the denatured heavy chain (IgG HC, 55 kDa) and light chain (IgG LC, 22 kDa) fragments of the immunoprecipitating anti-bodies are indicated with arrowheads.

Premature hyperactivation of CDC25C in human cancer cells via phosphorylation on S214, as observed in cells overexpressing low molecular weight isoforms of cyclin E, has been associated with premature mitotic entry, deregulation of G_2_-M transition, abrogation of the NOC-mediated mitotic arrest, centrosome amplification with emergence of cells with supernumerary centrosomes, multipolar anaphase spindles, chromosome missegregation, and polyploidy due to a cytokinesis failure (Bagheri-Yarmand et al., 2010a, Bagheri-Yarmand et al., 2010b). The observed mitotic aberrations in SYK-deficient cells and cells treated with the SYK inhibitor PCT were reminiscent of the mitotic aberrations reported for cells with hyperactivation of CDC25C associated with absence of inhibitory S216 phosphorylation (Bagheri-Yarmand et al., 2010a, Bagheri-Yarmand et al., 2010b). This prompted the hypothesis that SYK may act as a negative regulator of CDC25C by controlling its phosphorylation at S216. Therefore, we next set out to determine if these two regulatory proteins physically and functionally interact with each other. We first examined if SYK co-localizes with the centrosomal regulatory protein CDC25C. As evidenced by the confocal merge images of human BT20 cells depicted in Fig. 6a & b, SYK and CDC25C are co-localized in cytosol and centrosomes during metaphase and anaphase. This spatial arrangement of SYK and CDC25C provides a basis for physical as well as functional interactions. In co-immunoprecipitation experiments, SYK immune complexes contained CDC25C and CDC25C immune complexes contained SYK (Fig. 6c), providing unprecedented biochemical evidence for an in vivo physical association between native SYK and CDC25C in human cells. The detected kinase bands represent kinase proteins specifically pulled down by immunoprecipitation or co-immunoprecipitation as no SYK or CDC25C proteins were detected by Western blot analysis when no primary anti-SYK or anti-CDC25C antibodies were added to the immunoprecipitation mixtures.

### 3.4 SYK Phosphorylates CDC25C on Serine 216

**Fig. 7:**
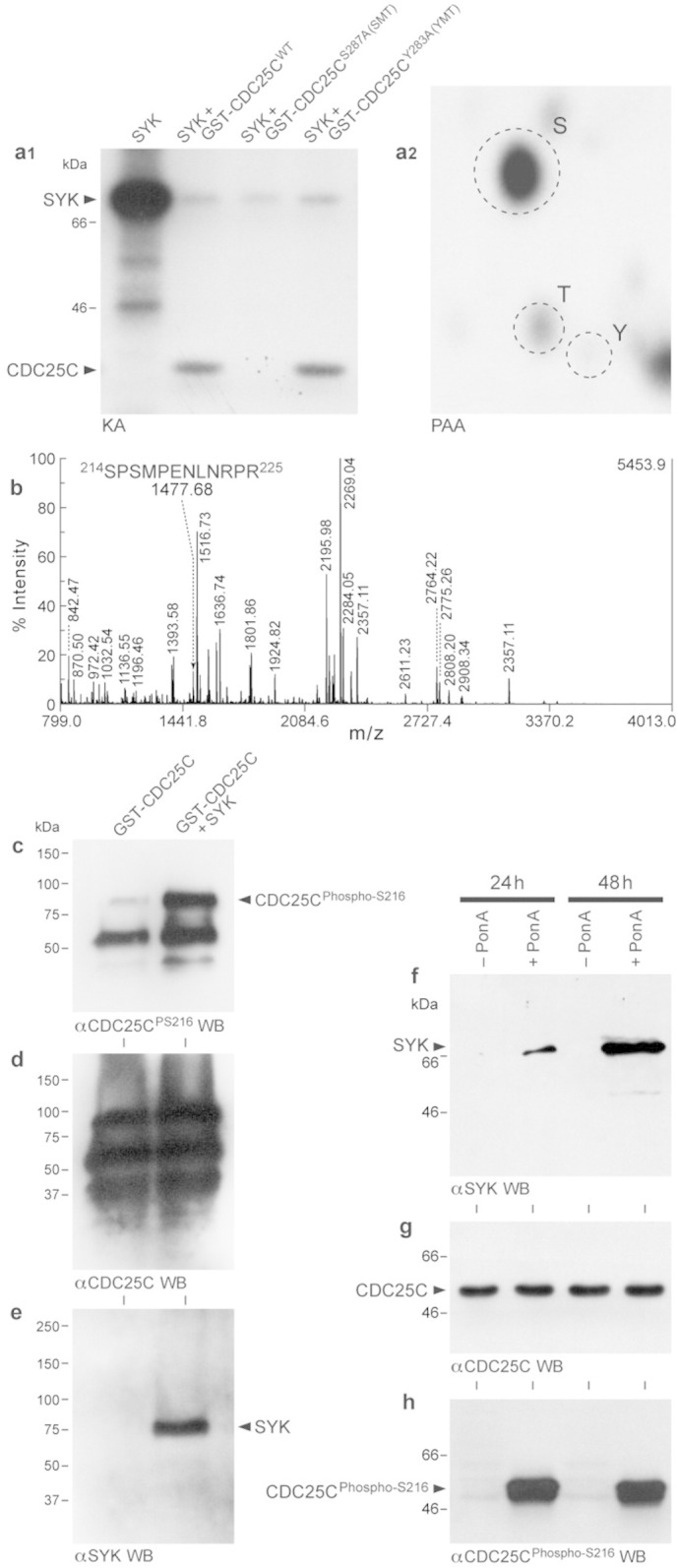
SYK phosphorylates CDC25C on serine 216. [a.1] In vitro phoshorylation of Serine 287 residue (corresponding to Serine 216 in human CDC25C) within the Xenopus GST-CDC25C peptide (254–316) by purified recombinant SYK. 1 mg of wildtype and mutant GST-CDC25C peptides were used as potential substrates in hot kinase assays using 1 μg recombinant murine SYK in the presence of 1 mCi of [γ-^32^P] ATP (Lanes 2–4). In control sample (Lane 1), 10 μg of recombinant SYK was allowed to autophosphorylate in the absence of GST-CDC25C peptide. The reaction mixtures were subjected to gel electrophoresis followed by autoradiography. Arrowheads indicate the positions of autophosphorylated SYK and phosphorylated GST-CDC25C peptides. Mutation of the S287 residue within the sequence of 254–316 of GST-CDC25C peptide to alanine abolished the phosphorylation detected on SYK-treated wildtype GST-CDC25C peptide (Lane 3 vs. Lane 2). By contrast, the mutation of Y283, which is the only tyrosine residue within the sequence of 254–316 of GST-CDC25C peptide to alanine did not affect its SYK-mediated phosphorylation (Lane 4). [a.2] Phospho amino acid analysis of the SYK phosphorylated wildtype GST-CDC25C peptide showed phosphorylation on serine. The ^32^P-labeled GST-CDC25C band in [A, Lane 2] was isolated and subjected to PAA. The positions of ninhydrin-stained phosphoamino acid standards (phosphoserine [S], phosphothreonine [T], and phosphotyrosine [Y]) are indicated with circles. [b] MALDI-TOF peptide MS of the tryptic digest of SYK-phosphorylated recombinant human CDC25C protein. The 1477.68 Da ion corresponds to single site (98 Da) phosphorylation of the ^214^SPSMPENLNRPR^225^ peptide containing the S216 residue that has one phosphate loss. Subsequent CID spectra revealed a peak at 1379.90 Da corresponding to a mass loss of 98 Da. [c–e] Purified recombinant SYK phosphorylates recombinant human full-length CDC25C protein on Serine 216. GST-tagged recombinant human CDC25C was treated with recombinant SYK in a cold kinase assay to determine if it can be phosphorylated by SYK. Western blot analysis of SYK-phosphorylated vs. control CDC25C protein with a phosphospecific antibody recognizing S216-phosphorylated CDC25C showed that recombinant SYK phosphorylates recombinant CDC25C on S216. The position of the S216-phosphorylated 83 kDa GST-tagged full-length CDC25C protein is denoted with an arrowhead (Panel c). Western blot analysis of the kinase assay mixtures with anti-CDC25C antibody showed near equal amounts of substrate protein in control and test samples. In addition to the intact 83 kDa protein, 55 kDa and 37 kDa proteolytic cleaved forms of GST-CDC25C are also seen in this commercial preparation (Panel d). The presence of SYK (predicted size: 72 kDa) in the kinase assay mixtures containing recombinant murine SYK was confirmed by anti-SYK Western blot analysis (Panel e). [f–h] In vivo S216 phosphorylation of native human CDC25C in an ecdysone-inducible mammalian expression system for human SYK. Panel f shows the anti-SYK Western blot analysis of whole cell lysates of untransfected vs. transfected U373 cells before and 24–48 h after exposure to the ecdysone-analogue Pon-A (10 μM). SYK protein (72–75 kDa, predicted size: 72 kDa) is detected in lysates of PonA treated cells. Panel g depicts the results of anti-CDC25C Western blot analysis of control SYK^−^ vs. Pon(A) treated SYK^+^ cells showing similar levels of native CDC25C protein (55 kDa, predicted size: 53 kDa) irrespective of SYK induction. Panel h shows that SYK induction causes S216 phosphorylation of native CDC25C protein, as detected by Western blot analysis using a highly specific antibody directed against the CDC25C phospho-epitope S216.

**Fig. 8:**
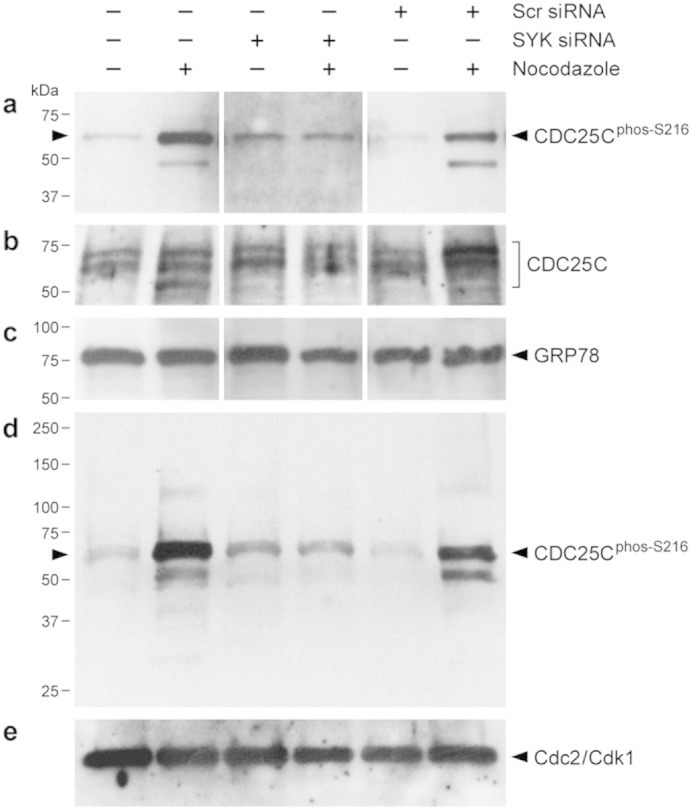
siRNA-mediated depletion of Native SYK prevents nocodazole-induced S216-phosphorylation of native CDC25C in human cells. [a–c] Anti-CDC25C(pS216), anti-CDC25C, and anti-GRP78 Western blot analysis of whole cell lysates from 293T cells treated with medium only (CON), SYK-siRNA or scr-siRNA that was used as a control. NOC treatment (0.1 μg/mL) for 24 h induced S216 phosphorylation of native CDC25C (Panel a, Lane 2 vs. Lane 1) without a change in the levels of CDC25C or GRP78 proteins (Lane 2 vs. 1, Panels b & c). SYK-siRNA abrogated NOC-induced S216 phosphorylation of CDC25C (Panel a, Lane 4 vs. Lane 3). By contrast, scr-siRNA did not block NOC-induced S216 phosphorylation of CDC25C (Panel a, Lane 6 vs. Lane 5). [d & e] Depicted are the results of a repeat analysis of SYK-siRNA effects on NOC-induced CDC25C phosphorylation on S216. Anti-CDC25C(pS216) (Panel d) and anti-cdc2/Cdk1 (Panel e) Western blot analysis of whole cell lysates from 293T cells treated with medium only (CON), SYK-siRNA or scr-siRNA that was used as a control showed that NOC treatment induced S216 phosphorylation of native CDC25C (Panel d, Lane 2 vs. Lane 1) without a change in the levels of Cdc2/Cdk1 (Panel e, Lane 2 vs. Lane 1). SYK siRNA abrogated NOC-induced S216 phosphorylation of CDC25C (Panel d, Lane 4 vs. Lane 3). By contrast, scr-siRNA did not block NOC-induced S216 phosphorylation of CDC25C (Panel d, Lane 6 vs. Lane 5). In a-e, each siRNA was used at a 50 nM concentration.

The primary phosphorylation site of CDC25C involved in G2 checkpoint control is at its S216 residue in humans and S287 residue in *Xenopus* (Perry and Kornbluth, 2007, Donzelli and Draetta, 2003, Kumagai and Dunphy, 1999). This residue is phosphorylated throughout interphase but not in mitosis and it is known to control the timing of mitosis. The S216 phosphorylated CDC25C binds to members of the 14-3-3 proteins and remains as a cytoplasmic complex (Kumagai and Dunphy, 1999). The cytoplasmic retention of CDC25C prevents it from activating CDK1 by Y15 and T14 dephosphorylation. We performed in vitro kinase assays to experimentally determine if recombinant SYK can phosphorylate the GST-tagged Xenopus CDC25C (245–316) peptide containing S287 that corresponds to the S216 residue of human CDC25C. This peptide also contains a single tyrosine residue (283 in *Xenopus* and 212 in human) located at the − 4 position with respect to S287 (YRSPSMP). SYK phosphorylated the wildtype CDC25C peptide and a phosphoaminoacid analysis of the SYK-phosphorylated CDC25C peptide showed that the phosphorylation occurred on serine (Fig. 7a.1 & a.2). Notably, recombinant SYK was able to phosphorylate the Y283A mutant form (“YMT”) of the CDC25C peptide but not its S287A mutant form (“SMT”) (Fig. 7a.1). We next performed in vitro kinase assays to determine if purified recombinant SYK can phosphorylate a GST-tagged full-length recombinant human CDC25C protein on S216 residue. A matrix-assisted laser desorption/ionization–time-of-flight (MALDI-TOF)/TOF mass spectrometry analysis was performed on trypsin-digested recombinant CDC25C after an in vitro kinase reaction with recombinant SYK. After TiO_2_ enrichment, a precursor with a mass of 1477.68 Da corresponding to the peptide ^214^SPSMPENLNRPR^225^ containing the S216 phosphoepitope in phoshorylated form was identified (Fig. 7b). The Western blot analysis of the CDC25C protein from the SYK plus CDC25C kinase reaction mixtures with a phosphospecific antibody that recognizes S216-phosphorylated CDC25C showed markedly enhanced S216 phosphorylation (Fig. 7c–e). Having confirmed the ability of recombinant purified SYK to phosphorylate CDC25C on the inhibitory residue S216 in vitro, we next examined the regulatory role of SYK in CDC25C phosphorylation in vivo using an ecdysone-inducible mammalian expression system (Uckun et al., 2010a). Pon-A exposure of SYK-deficient U373 cells stably transfected with wildtype human *syk* gene induced expression of SYK within 24 h (Fig. 7f). SYK induction without any exposure to NOC was sufficient for activating S216-phosphorylation of CDC25C, as determined by Western blot analysis using a CDC25C^phos-S216^ specific antibody (Fig. 7h). We next examined the effects of RNAi-mediated SYK depletion on the NOC-triggered S216 phosphorylation of CDC25C in 293T cells. Treatment of human 293T cells with NOC triggered phosphorylation of CDC25C on the inhibitory S216 residue (Fig. 8). SYK siRNA abrogated the NOC-induced S216-phosphorylation of CDC25C, whereas treatment with scrambled siRNA (included as a negative control) had no such effect (Fig. 8 a & d). These results demonstrate that SYK is required for S216 phosphorylation of native CDC25C in vivo after NOC exposure and provide compelling support for the notion that native CDC25C in human cells is not only physically associated with SYK, but it also serves as an in vivo kinase substrate for native SYK.

**Fig. 9:**
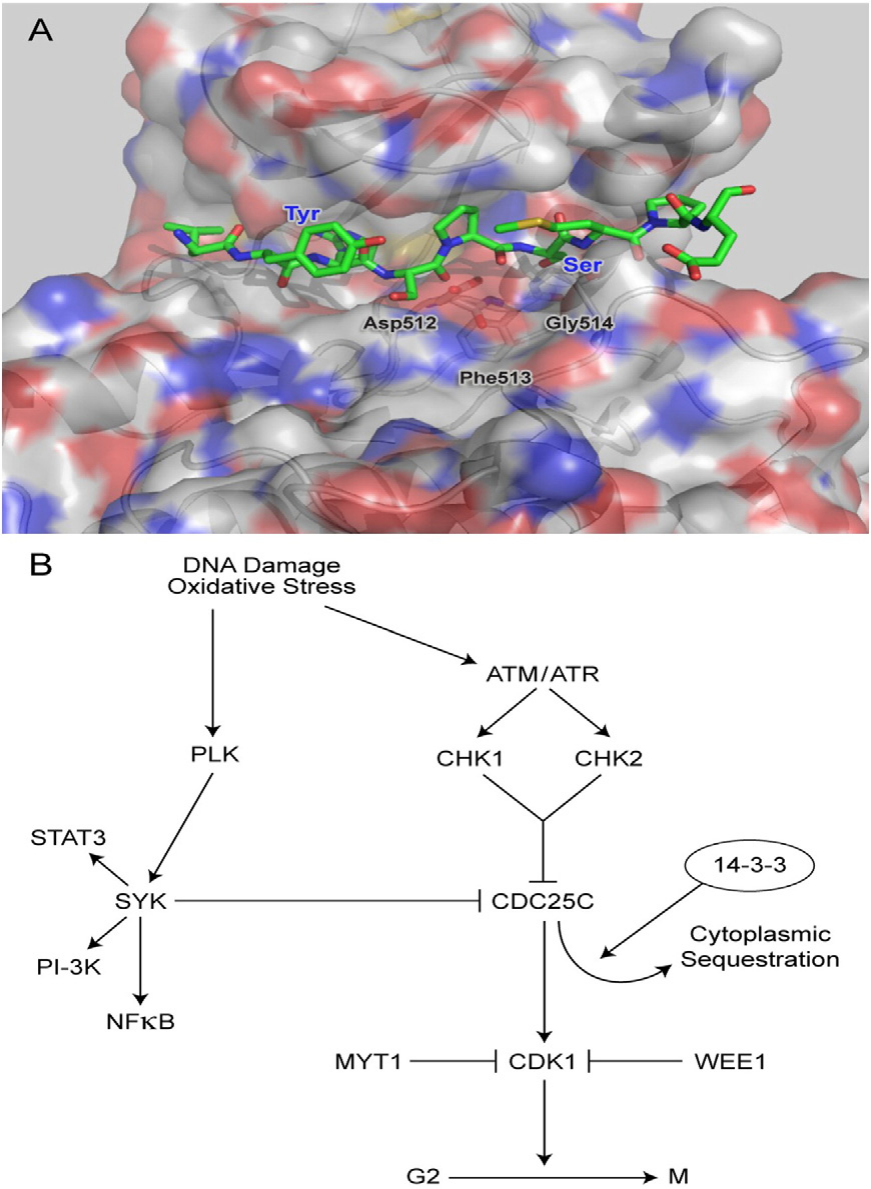
SYK as a negative regulator of CDC25C [A] molecular surface of SYK kinase with modeled CDC25C peptide. The DFG motif in catalytic site of SYK (Asp512-Phe513-Gly514) is shown as stick model. The molecular surface of SYK is colored by atom type: carbon in gray, oxygen in red, nitrogen in blue, and sulfur in yellow. Substrate peptide (Leu-Tyr-Arg-Ser-Pro-Ser-Met-Pro-Glu, residues 211–219 of human CDC25C) is shown as stick model and color-coded by atom type: carbon, green; nitrogen, blue; oxygen, red; sulfur, yellow. The figure was prepared with PyMOL Molecular Graphics System (version ro.99, Schrodinger LLC). According to this model, the 9-amino acid CDC25C peptide binds in an extended conformation to the catalytic groove on the surface of SYK and interacts with its N- and C-terminal subdomains. The Ser216 residue of the substrate CDC25C peptide is hydrogen bonded with the conserved Asp512 residue of SYK that serves as the catalytic base to accept the proton from Ser216. The Ser214 and Arg213 residues of the CDC25C peptide form hydrogen bonds with the Arg498 and Ser511 residues of SYK. [B] A model of Cell cycle regulation in the context of oxidative stress and DNA damage. In response to oxidative stress or DNA damage, the ATM/ATR signaling pathway is activated leading to downstream activation of CHK1 and CHK2 kinases that phosphorylate CDC25C on S216. S216 phosphorylation of CDC25C has been shown to inhibit its CDK1-activating function in the nucleus by enhancing its binding to 14-3-3 proteins and thereby causing its sequestration in the cytoplasm. We propose that a negative feedback loop exists between PLK and SYK that rapidly limits the pro-mitotic signal of PLK by inhibitory S216 phosphorylation of CDC25C by PLK-activated SYK in cells exposed to oxidative stress. This previously unrecognized function of SYK may serve as a physiologically important backup regulatory surveillance system for DNA damage and complement the functions of other checkpoint regulators by preventing the reinitiation of DNA synthesis before the mitosis is correctly completed or DNA damage is repaired.

We constructed a structural model of a complex between SYK and the CDC25C peptide Leu-Tyr-Arg-Ser-Pro-Ser-Met-Pro-Glu (residues 211–219) to evaluate the molecular mechanism for the ability of SYK to phosphorylate CDC25C on S216. This model posits that the target CDC25C peptide would readily bind to SYK catalytic site with a compact conformation due to its narrow and deep shape (Fig. 9A). Because of space constraints, the small S216 residue is more likely to fit the hydrophobic pocket created by the activation loop containing the DFG motif (Asp512-Phe513-Gly514) than the larger Y212 residue.

## 4 Discussion

The activity of centrosomal CDK1 plays a crucial role in the regulation of mitotic timing. RNAi-mediated depletion of centrosomal CDK1 or CEP63 that recruits CDK1 to centrosomes causes accumulation of “giant cells” due to polyploidization through mitotic skipping (Löffler et al., 2011). Before mitosis, CDK1 is kept in an inactive state via phosphorylation at T14 and Y15, which is catalyzed by the protein kinases WEE1 and MYT1 (Lindqvist et al., 2009). CDK1 activation, on entry into mitosis, results from simultaneous inhibition of WEE1 and MYT1 and activation of CDC25C. Any corruption of this regulatory process of activation and inactivation of CDK1 can trigger mitotic defects. The G_2_ checkpoint prevents CDC25C phosphatase from removing the T14/Y15 phosphate groups on CDK1 and thereby provides more time for DNA damage repair prior to mitotic entry. This is accomplished by maintaining CDC25C in an inactive S216-phosphorylated form. Our findings presented herein provide the first genetic and biochemical evidence for a previously unknown function of SYK as a cell cycle regulatory kinase that phosphorylates CDC25C at S216. This unique role of SYK as a cell cycle checkpoint regulator may represent a significant challenge for SYK inhibitors that are being developed for various indications.

Homozygous CDC25C knockout (CDC25C^−/−^) mice are viable, fertile, develop normally and do not have an obvious phenotype (Chen et al., 2001, Lee et al., 2011). These findings indicate that the related protein phosphatases CDC25A and/or CDC25B may compensate for loss of CDC25C in the mouse (Chen et al., 2001, Lee et al., 2011). However, overexpression or hyperactivation of CDC25C alone is sufficient to cause mitotic aberrations. In particular, a premature activation of CDC25C by S214 phosphorylation, which renders it resistant to inhibitory S216 phosphorylation, can cause dephosphorylation of CDK1 on T14 and Y15, thereby activating CDK1 and promoting premature mitotic entry (Bagheri-Yarmand et al., 2010b). Premature hyperactivation of CDC25C in human cancer cells via phosphorylation on S214, as observed in cells overexpressing low molecular weight isoforms of cyclin E, has indeed been associated with premature mitotic entry, deregulation of G_2_-M transition, abrogation of the NOC-mediated mitotic arrest, emergence of centrosome amplification with emergence of cells with supernumerary centrosomes, multipolar anaphase spindles, chromosome missegregation, and polyploidy due to a cytokinesis failure (Bagheri-Yarmand et al., 2010b). CDC25C has been shown to mediate these mitotic aberrations because they are abrogated by RNAi-induced depletion of CDC25C (Bagheri-Yarmand et al., 2010b). Notably, cells rendered SYK-deficient by homologous recombination knockout or RNAi of the SYK gene as well as functionally SYK-deficient cells treated with the SYK inhibitor PCT displayed G_2_ checkpoint abnormalities reminiscent of the aforementioned mitotic aberrations reported for cells with CDC25C hyperactivated due to resistance to S216 phosphorylation. In particular, SYK-specific siRNA as well as SYK inhibitor PCT were effective in overriding a checkpoint-dependent mitotic arrest provoked by NOC treatment in both B-lineage lymphoid and non-lymphohematopoietic human cells. The documented ability of SYK to phosphorylate CDC25C on S216 provided a cogent explanation for this phenotype associated with SYK-deficiency and uniquely indicated that other kinases capable of S216 phosphorylation, are unable to compensate for SYK deficiency. SYK thus appears to be an essential component of a cell cycle regulatory surveillance system in human cells.

Human polo-like kinase (PLK) physically associates with SYK at the mitotic spindle poles (Uckun et al., 2010b). In the context of oxidative stress, PLK has been shown to activate SYK by phosphorylating it on T524 residue, which is located in a critical position on the turn of the hairpin structure of the activation (A)-loop of the SYK kinase domain in close relationship to the activation residues Y519/Y520 (Uckun et al., 2010b). According to our previously reported molecular modeling studies, PLK-induced phosphorylation of SYK on T524 would unlock the tangled inhibitory conformation of A-loop and promote the phosphorylation of the activation residues Y519/Y520 (Uckun et al., 2010b). The physiologic role of PLK-induced SYK activation may be to enhance the survival of cells challenged with oxidative stress-associated DNA damage by evoking an anti-apoptotic response via activation of SYK and SYK-dependent NFκB, PI3-K/AKT, and STAT3 signal transduction pathways (Uckun and Qazi, 2010). As an M-phase specific serine/threonine kinase, PLK regulates CDK1 function via phosphorylation and activation of CDC25C (Wang et al., 2009, Bonni et al., 2008, Roshak et al., 2000, Xie et al., 2005, Bahassi et al., 2004). CDC25C is predominantly cytoplasmic and its nuclear import is triggered by PLK-induced phosphorylation of S191 and S198 residues within an N-terminal functional nuclear exclusion motif (Wang et al., 2009, Roshak et al., 2000, Xie et al., 2005, Bahassi et al., 2004). We propose that a negative feedback loop exists between PLK and SYK that rapidly limits the pro-mitotic signal of PLK via inhibitory S216 phosphorylation of CDC25C by PLK-activated SYK in cells exposed to oxidative stress (Fig. 9B). The previously unrecognized function of SYK as a regulator of G_2_ checkpoint may serve as a physiologically important backup regulatory surveillance system for DNA damage and complement the functions of other checkpoint regulators by preventing the reinitiation of DNA synthesis before the mitosis is correctly completed or DNA damage is repaired. The presented evidence for the existence of a cell cycle checkpoint regulatory function of SYK that controls DNA replication extends previous studies respecting the involvement of protein kinases in cell cycle regulation as well as studies on the pleiotropic biologic effects of SYK kinase-linked biochemical signals.

Spleen tyrosine kinase (SYK) is a physiologically important kinase that serves as a key regulator of multiple biochemical signal transduction events and biologic responses in B-lineage lymphoid cells throughout B-cell ontogeny (Cheng et al., 1995, Mocsai et al., 2010, Turner et al., 1997, Uckun and Qazi, 2010, Zhou et al., 2006). SYK deficiency is associated with pro-B cell leukemia in infants (Goodman et al., 2001). Recently SYK has been identified as a dual-specificity kinase that not only phosphorylates tyrosine but also serine (S) residues (Heizmann and Reth, 2010). SYK also has important functions in BCR-independent signaling pathways in lymphohematopoietic as well as non-lymphohematopoietic cells (Uckun and Qazi, 2010, Zhou et al., 2006, Goodman et al., 2001, Heizmann and Reth, 2010, Wang et al., 2005, Uckun et al., 2010a, Uckun et al., 2012). As a regulatory tyrosine kinase, SYK plays an important and indispensable role in oxidative stress-induced activation of the anti-apoptotic transcription factor STAT3 via tyrosine phosphorylation and its catalytic domain is critical for this survival-promoting function (Uckun et al., 2010a). Homozygous *syk* knockout mice suffer severe hemorrhaging as embryos and virtually all die at midgestation or perinatally due to lymphatic hyperproliferation, vascular defects and blood-lymphatic shunts (Cheng et al., 1995, Böhmer et al., 2010, Abtahian et al., 2003). SYK tyrosine kinase has also been identified as a mitotic kinase that localizes to the centrosomes (Uckun et al., 2010b, Navara et al., 1999, Xue et al., 2012, Zyss et al., 2005) and affects mitotic progression (He et al., 2002, Zyss et al., 2005). Several candidate centrosomal substrates for SYK were identified by using sensitive kinase assays linked with phosphoproteomics, supporting a unique mechanism whereby SYK negatively affects cell division through its centrosomal kinase activity (Xue et al., 2012).

The activity of centrosomal CDK1 plays a crucial role in the regulation of mitotic timing. RNAi-mediated depletion of centrosomal CDK1 or CEP63 that recruits CDK1 to centrosomes causes accumulation of “giant cells” due to polyploidization through mitotic skipping (Löffler et al., 2011). Before mitosis, CDK1 is kept in an inactive state via phosphorylation at T14 and Y15, which is catalyzed by the protein kinases WEE1 and MYT1 (Lindqvist et al., 2009). CDK1 activation, on entry into mitosis, results from simultaneous inhibition of WEE1 and MYT1 and activation of CDC25C. Any corruption of this regulatory process of activation and inactivation of CDK1 can trigger mitotic defects. The G_2_ checkpoint prevents CDC25C phosphatase from removing the T14/Y15 phosphate groups on CDK1 and thereby provides more time for DNA damage repair prior to mitotic entry. This is accomplished by maintaining CDC25C in an inactive S216-phosphorylated form. Our findings reported herein provided both genetic and biochemical evidence for a previously unknown function of SYK as a cell cycle regulatory kinase that phosphorylates CDC25C at S216. Hence, SYK was discovered as an inhibitor of CDC25C which prevents premature entry into mitosis by phosphorylating CDC25C at S216 when G_2_ checkpoint responses are activated. Notably, SYK induction was associated with marked changes in the cell cycle related transcriptome. Gene set enrichment analysis showed that SYK induction in human cells causes a significant down-regulation of evolutionarily conserved genes associated with mitosis and cell cycle progression, indicating that SYK induction likely delays the mitotic entry. The down-regulated genes in SYK-induced cells included the human homologs of 3 yeast genes (viz.: *CDC20*, *DBF4*, *BUB3*) previously demonstrated to have peak expression in the M phase of the yeast cell cycle (Rowicka et al., 2007). Inactivation of CDC25C by ATM signaling triggered by DNA damage (Innes et al., 2006, Stracker et al., 2008) has been shown to be associated with downregulation of a unique group of highly correlated G_2_ checkpoint signature genes and those genes are upregulated in ATM-deficient mouse cells. Notably, the human homologs of these genes were also downregulated by induction of SYK expression in human cells. The down-regulation of the human orthologs of yeast M-phase genes and human orthologs of ATM-dependent murine G_2_-checkpoint genes as well as ATM-dependent human radiation-response genes supports the model that SYK induction activates a G_2_ checkpoint and thereby delays mitotic entry.

The identification of SYK as a master regulator of anti-apoptotic signaling in B-lineage leukemia/lymphoma cells prompted the development of SYK inhibitors as a new class of anti-cancer drug candidates (Uckun and Qazi, 2010, D'Cruz and Uckun, 2012, Myers et al., 2014, Uckun et al., 2013, Perova et al., 2014, Wang et al., 2014, Geahlen, 2014). However, the discovery that SYK is a dual-function kinase with important tumor suppressive and cell cycle regulatory roles represents a new and unexpected challenge for the translational research efforts. SYK has been previously shown to play an important tumor suppressor function during human lymphocyte ontogeny by protecting the lymphoid progenitors from a leukemogenic CK2-mediated inhibition of Ikaros function (Uckun et al., 2012). Furthermore, SYK-induced serine phosphorylation is required for the ability of IK to mediate the differentiation of human B-cell precursors (Uckun et al., 2012). Indeed, defective SYK expression has been implicated in the pathogenesis of infant pro-B cell acute lymphoblastic leukemia (ALL) (Goodman et al., 2001), which is thought to originate from B-cell precursors with a maturational arrest at the pro-B cell stage and is associated with poor prognosis. Notably, B-cell precursors from infant patients with pro-B cell leukemia have markedly reduced SYK activity due to expression of defective SYK proteins with a missing or truncated catalytic kinase domain coded by profoundly aberrant mRNA species (Goodman et al., 2001). This association between SYK deficiency and development of aggressive pro-B cell leukemia in infancy may be caused by a loss of SYK-induced phosphorylation of IK on activating serine residues S358 and S361 (Uckun et al., 2012). Consequently, the use of kinase inhibitors of the conserved ATP binding site within the catalytic domain of SYK, which is required for both its tyrosine kinase activity and serine kinase activity, as are most SYK inhibitors in preclinical or clinical development (D'Cruz and Uckun, 2012, Perova et al., 2014, Geahlen, 2014), including compound R406 and its pro-drug R788 (Fostamatinib disodium/FosD), may contribute to an increased risk of emergence of new leukemic clones and progression of leukemia, especially in pediatric leukemia patients who are subjected to DNA damaging agents as part of their multi-modality standard treatment programs. Furthermore, because of the similarities of the ATP pocket structures among different kinases, most of these inhibitors affect multiple tyrosine kinases and have off-target activities (D'Cruz and Uckun, 2012). Indeed hypertension, a common and potentially dangerous side effect of FosD, has been attributed to off-target inhibition of VEGFR (D'Cruz and Uckun, 2012). Inhibitors targeting the substrate binding sites of tyrosine kinases are hoped to have enhanced specificity and potency (Uckun et al., 2010a, Myers et al., 2014, Uckun et al., 2013). The selective inhibition of anti-apoptotic tyrosine phosphorylation events by blocking the binding of the substrates of SYK (rather than inhibiting the ATP binding site) would not cause a malfunction of Ikaros because it spares the ATP site-dependent serine kinase function of SYK. Therefore, it will be very important to develop selective inhibitors of the tyrosine kinase substrate binding (P)-site of SYK.
