# Supplementary material for: A Previously Unknown Unique Challenge for Inhibitors of SYK ATP-Binding Site: Role of SYK as A Cell Cycle Checkpoint Regulator
Source: eBioMedicine. 2014 Oct 30;1(1):16–28. doi: 10.1016/j.ebiom.2014.10.019 (PMC4259291; doi:10.1016/j.ebiom.2014.10.019)
Supplement: Supplementary file 1 — Supplementary material. [file mmc1.doc]

**SUPPLEMENTAL MATERIAL**

**Materials and Methods**

**Cells.** An EBV-transfected B-lymphoblastoid cell line from our laboratory (BLCL-1) was grown in RPMI (Life Sciences, Frederick, MD) supplemented with 10% fetal bovine serum and 1.0% Penicillin and Streptomycin. We also used the following human cell lines: the human embryonic kidney cell line 293T (ATCC®, CRL-11268), human breast cancer cell line BT20 (ATCC®, HTB-19) human glioblastoma cell line U373 (ATCC®, HTB-17) and its SYK^+^ subclone U373-PHC, LOUCY (T-lineage ALL; ATCC® CRL-2629™)^1-3^. In addition, the wildtype DT40 chicken B lymphoma cell line, its SYK-deficient subclone established by homologous recombination knockout of the *syk* gene, and SYK-deficient DT40 cells reconstituted with wildtype *syk* were used as components of a well-established genetic model for B-lineage lymphoid cells^1,2^.

**Establishment of a Human Cell Line Expressing EYFP-Tagged α-Tubulin.** The *pEYFP-Tub* vector designed for expressing the EYFP-tubulin fusion protein in mammalian cells was purchased from Clontech (Mountain View, CA). *pEYFP-Tub* encodes a fusion protein consisting of enhanced yellow fluorescent protein (EYFP) and human α-tubulin. EYFP is an enhanced yellow-green variant of the Aequorea victoria green fluorescent protein (GFP). The EYFP protein contains the four amino acid substitutions Ser-65 to Gly; Val-68 to Leu; Ser-72 to Ala; and and Thr-203 to Tyr as well as the Phe-64 to Leu mutation of GFPmut1. The fluorescence excitation maximum of EYFP is 513 nm; the emission spectrum has a peak at 527 nm (in the yellow-green region). The fusion protein incorporates directly into microtubules in transfected cells and thereby allows the observation of microtubules by fluorescence microscopy. The construct was linearized with the *AseI* restriction enzyme (New England Biolabs, Ipswich, MA). SYK-positive U373-PHC human glioblastoma cells (1 x 10^7^) were transfected with 12 μg of pEYFP-*Tub* at 240 V / 950 µF using a BioRad GenePulser II Electroporator (Biorad, Hercules, CA). Cells were then reseeded in 150 x 15 mm tissue culture dishes and stably transfected cells were selected with 500 μg∕mL G418-sulfate (Neomycin, Gibco BRL) and isolated by standard fluorescence activated cell sorting using a FACS Vantage Instrument (Becton Dickinson, San Jose, CA)^3^.

**Establishment of U373 Cells with Ecdysone-Inducible SYK Gene Expression.** SYK-deficient U373 cells were transfected with the ecdysone inducible system regulatory vector, pVgRXR, and with a pIND/GS vector containing the cDNA encoding wildtype human *syk*

gene (H-L28824MI) (Invitrogen) using published procedures^1^. pIND-*syk* was linearized with AatII and pVgRXR was linearized with MIuI. Ten million U373 cells were transfected with 12 μg of each linearized vector by electroporation using a BioRad GenePulser II Electroporator (240 V∕950 μF). Two days after electroporation, cells were reseeded in 150x15 mm tissue culture dishes and stably transfected cells were selected with 500 μg∕mL G418-sulfate and 500 μg∕ml zeocin. Individual clones were screened for the inducible expression of SYK after 24 h treatment with 10 μM ponasterone (Pon-A) (an analogue of ecdysone) (Invitrogen) by Western blot analysis^1^. Pon-A Exposure of SYK-deficient U373 cells stably transfected with wildtype *syk* gene induces expression of SYK and activates downstream signaling events mimicking oxidative stress-induced activation of SYK and SYK-dependent signal transduction pathways^1^.

**Establishment of a Human Cell Line Expressing GFP-Tagged SYK for Subcellular Localization Studies by Confocal Imaging.** The rat cDNA coding for the full-length SYK protein^4^ was cloned into the 4.7 kb parent vector pEGFP-N1 according to the manufacturer’s guidelines (GenBank accession number U55762, Clontech Laboratories) to construct the recombinant eukaryotic expression vector pEGFP-*SYK*. U373 human glioblastoma cells (1x10^7^) were transfected with linearized pEGFP-*SYK* plasmid (12 µg) by electroporation at 240 V/950 µF using the Gene Pulser II (Bio Rad, Hercules CA). Cells were reseeded in 150x15 mm tissue culture dishes and subjected to G418 (500 µg/mL; Gibco, BRL) selection 48 h later. By using fluoresence microscopy for screening, stably transfected U373 cells expressing GFP-tagged recombinant rat SYK protein were selected by subcloning. Western blot analysis of whole cell lysates of selected U373 cells stably transfected with pEGFP-*SYK* using anti-SYK or anti-GFP antibodies confirmed the expression of a GFP-SYK fusion protein of M(r) 99,000 which consists of SYK and GFP fused to the N-terminus of SYK. In kinase assays, whole cell extracts of pEGFR-*SYK* transfected U373 cells were immunoprecipitated with anti-GFP monoclonal antibody. Immunoprecipitates were washed in kinase buffer (2 mM Tris, 5mM MgCl_2_, 5mM MnCl_2_) and incubated with the SYK substrate GST-Igα in the presence of ^32^P-ATP at 25°C for 20 min using previously published kinase assay procedures^1,2,5,6^.

Subcellular co-localization studies using immunofluorescence and confocal microscopy were performed as previously described^1,2^. Cells were plated onto glass coverslips either immediately before fixation (BLCL1) or 24 h before fixation (BT20 and U373 cells) and fixed with ice-cold methanol for 10 min. Cells were then placed in PBS + 0.1% Triton X-100(Tx) (PBS + Tx) for 15 min before labeling. Primary antibodies were diluted in PBS + Tx and incubated on the coverslips for 40 min at 37° C in a humidified chamber. After a 15 min wash in PBS+ Tx an appropriate fluorescently labeled secondary antibody was added to the coverslips for an additional 40 min. After another 15 min wash in PBS + Tx, 5mM TOTO-3 (Molecular Probes, Eugene OR) was added for 20 min to label the nuclear DNA. Coverslips were mounted in vectashield mounting medium to prevent photobleaching. Slides were sealed with nail polish. Slides were examined using a Bio-Rad MRC 1024 Laser Scanning Confocal Microscope equipped with a Kr/Ar laser (Bio-Rad, Hercules CA) attached to a Nikon Eclipse E-800 microscope with high numerical aperture objectives (Nikon, Melville, NY). U373 cells transfected with the pEGFP-*SYK* plasmid were also imaged using deconvolution microscopy on a Deltavision (Applied Precision Inc. Seattle WA) deconvolution microscope.

**Reagents**. The rabbit polyclonal C-20 (sc-929) antibody reactive with the C-terminus of human SYK, rabbit polyclonal N-19 (sc-1077) antibody reactive with the N-terminus of human SYK, mouse monoclonal 4D10 (sc-1240) antibody reactive with human SYK and weakly with ZAP70, the mouse monoclonal anti-α-tubulin antibody TU-02 (sc-8035), the mouse monoclonal antibody anti-Cdc25(TC-14) (sc-56269), as well as the rabbit polyclonal anti-phospho-Cdc25C(Ser216) antibody (sc-12354) were obtained from Santa Cruz Biotechnology, Inc. (Santa Cruz Biotechnology, CA). The mouse monoclonal anti-γ-Tubulin (T6557) antibody was purchased from Sigma (St. Louis, MO, USA). The mouse monoclonal anti-BiP/GRP78 antibody (Cat. No. 610978) was purchased from BD Biosciences (San Jose, California). Mouse monoclonal anti-GFP antibody was obtained from Clontech (Catalog No. 832-1) (Mountain View, CA). Nocadozole (M1404) (NOC) was obtained from Sigma (St. Louis, MO, USA). Goat anti-mouse IgG: Horseradish Peroxidase (HRPO) (M15345) and goat anti-rabbit IgG: HRPO (R14745) antibodies were purchased from Transduction Labs. UltraCruz™ Mounting Medium containing 1.5 μg/ml of 4', 6-diamidino-2-phenylindole (DAPI) was purchased from Santa-Cruz Biotechnology, Inc. (sc-24941). TOTO-3 iodide was obtained from Molecular Probes (Eugene, OR). Molecular weight markers were purchased from Amersham Pharmacia Biotech. Protein A-Sepharose was purchased from Repligen Corp. (Cambridge, MA). Restriction enzymes and proteinase inhibitors were purchased from Roche (Indianapolis, IN). The siRNA pool used for knock-down of the *SYK* gene (Gene ID: 6850) (Catalog No. L-003176) included 5’-GCUGCGCAAUUACUACUAU-3’, 5’-GAGCAAAUUGUCCUGAUAG-3’, 5’-CGGAAUGCAUCAACUACUA-3’, and 5’-AGAAAUGUGUUGCUAGUUA-3’^2^. The ON-TARGET*plus* Non-Targeting siRNA scrambled control pool (Catalog No. D-001810) was used as a control. All siRNA were purchased from Thermo Scientific Dharmacon, Lafayette, CO, USA.

**Microscopy.** Confocal laser scanning microscopy was performed as previously described^1,2,5^. Cells were examined using a Bio-Rad MRC-1024 Laser Scanning Confocal Microscope equipped with a Krypton(Kr)/Argon (Ar) laser (BioRad, Hercules, CA) (excitation at 488, 568, 647nm) mounted on a Nikon Eclipse E800 upright microscope with high numerical aperture objectives (Nikon, Melville, NY) and epi-fluorescence optics with the necessary fluorescence excitation and emission filter combinations. Digital images of fixed cells were captured with a Princeton Instruments MicroMax CCD camera (model RTE/CCD-1300-Y/HS, Roper Scientific, Trenton, New Jersey) using MetaMorph imaging software (Universal Imaging, Downingtown, Pennsylvania). In some experiments, cells were seeded onto sterile 22 mm^2^ coverslips in six-well plates. Cells on coverslips were returned to the incubator for 24 h prior to treatment. The following day PCT, NOC or NOC+ PCT was added to yield the indicated final concentrations and cells were returned to a 37^o^C/5% CO_2_ incubator for 48 h. Cells on coverslips were extracted prior to fixation by a 10 min treatment with 4% CHAPS detergent in 1.0X PHEM buffer (60 mM PIPES, 25 mM HEPES, 10 mM EGTA, 2 mM MgCl_2_, pH 6.95). Cells were then fixed for 10 min in 1.0XPHEM buffer+0.15% glutaraldehyde and 2% formaldehyde. This was followed by a 15 min incubation in PBS+0.1% Triton X-100 (PBS+Tx). Coverslips were next incubated for 40 min at 37^o^C with a primary antibody recognizing α-Tub (Sigma, St Louis, MO) in a humidifed chamber. Coverslips were washed for 15 min in PBS-Tx followed by a 40 min incubation with a fluorescently labeled secondary antibody (Jackson Immunoresearch, West Grove, PA). No primary or secondary antibodies were used for U373-PHC cells expressing green-fluorescent EYFP-tagged α-Tub. The coverslips were again rinsed in PBS-Tx and incubated with 5 mM DAPI (Sigma, St Louis, MO) for 20 min to label the nuclear DNA. Coverslips were immediately inverted onto slides in Vectashield (Vector Labs, Burlingame, CA) to prevent photobleaching, sealed with nail polish and stored at 4^o^C. Slides were examined using a Nikon TE200 inverted microscope with high numerical aperture objectives equipped for phase contrast and epifluorescence imaging^7^. Digital images were collected with a Princeton Instruments Micromax digital camera using Metamorph software (Universal Imaging, West Chester, PA). One-way Analysis of Variance (ANOVA) models with Dunnett's post-hoc tests were utilized to compare differences between each of the 3 treatments performed in triplicate (NOC, PCT, NOC+PCT) against the mean of the control group for % of cells with mitotic aberrations.

Subcellular co-localization studies using immunofluorescence and confocal microscopy were performed as previously described^1,2^. Cells were plated onto glass coverslips either immediately before fixation (BLCL1) or 24 h before fixation (BT20 and U373 cells) and fixed with ice-cold methanol for 10 min. Cells were then placed in PBS + 0.1% Triton X-100(Tx) (PBS + Tx) for 15 min before labeling. Primary antibodies were diluted in PBS + Tx and incubated on the coverslips for 40 min at 37° C in a humidified chamber. After a 15 min wash in PBS+ Tx an appropriate fluorescently labeled secondary antibody was added to the coverslips for an additional 40 min. After another 15 min wash in PBS + Tx, 5mM TOTO-3 (Molecular Probes, Eugene OR) was added for 20 min to label the nuclear DNA. Coverslips were mounted in vectashield mounting medium to prevent photobleaching. Slides were sealed with nail polish. Slides were examined using a Bio-Rad MRC 1024 Laser Scanning Confocal Microscope equipped with a Kr/Ar laser (Bio-Rad, Hercules CA) attached to a Nikon Eclipse E-800 microscope with high numerical aperture objectives (Nikon, Melville, NY). U373 cells transfected with the pEGFP-*SYK* plasmid were also imaged using deconvolution microscopy on a Deltavision (Applied Precision Inc. Seattle WA) deconvolution microscope.

During confocal imaging in other experiments, slides were imaged using the PerkinElmer Spinning Disc Confocal Microscope and the PerkinElmer UltraView ERS software (Shelton, CT) or the Volocity V5.4 imaging software (PerkinElmer, Shelton, CT)^2^. Cells were stained with appropriate primary and secondary antibodies, using previously reported procedures^1,2^. Cells were then washed with PBS and counterstained with the DNA-specific nuclear dye 4’,6-diamidino-2-phenylindole (DAPI). The coverslips were inverted, mounted onto slides in Vectashield (Vector Labs, Burlinghame, CA) to prevent photobleaching, and sealed with nail varnish. UltraCruz Mounting Medium containing 1.5 μg/ml DAPI was purchased from Santa Cruz Biotechnology, Inc. (Santa Cruz, CA)^2^. In NOC challenge experiments using 293T cells, cells were transfected with the respective siRNA (SYK siRNA vs. scrambled siRNA) at 50 nM concentration for 72 h and then cultured in the presence of 0.12 µg/mL (400 nM) NOC for another 48 h. SYK/DAPI stained cells were examined by confocal microscopy at 48 h post NOC exposure (i.e., 120 hours after start of siRNA exposure). Light microscopy images of Wright-Giemsa stained DT40 cells were taken using a LEICA DM LB microscope (Houston, TX) equipped with a Sony NEX-3 camera system.

**DNA Flow Cytometry.** Cells (5x10^5^ per mL in plastic tissue culture flasks) were treated with NOC (0.12 µg/mL x 48 h at 37^o^C), washed at 48 h, and then examined by DNA flow cytometry for emergence of polyploid cells at 48 h or 72 h, as described^6^. Propidium iodide (PI, Sigma) was used to determine the percentages of cells in each phase of the cell cycle by quantitative DNA flow cytometry. Plasma membranes were lysed in a hypotonic solution of 50 µg/mL PI in 0.1% sodium citrate, 1 mM Tris (pH 7.4), 0.1 mM EDTA, and 0.1% Triton-X-100 for 30 min in the dark at 4^o^C. DNA histograms of cells were derived from the flow cytometric acquisition of 20,000 events and the fraction of cells in each phase of the cell cycle was determined using a FACStar Plus flow cytometer (Becton Dickinson, San Jose, CA) with a 488 nm excitation from an argon laser.

**Recombinant SYK and CDC25C Proteins.** Recombinant murine SYK was produced in a baculovirus expression system and purified as previously described in detail^1,2^. GST-tagged full-length recombinant human CDC25C (Approximate molecular weight: 83 kDa) (Catalog no. C04-20CG, Lot. No. H140-1) produced in a baculovirus expression system was purchased from SignalChem (Richmond, BC, Canada). In addition to the intact 83 kDa protein, 55 kDa and 37 kDa proteolytic cleaved forms of GST-CDC25C are also detected in this commercial preparation. Plasmids for GST-tagged wildtype Xenopus CDC25 (254-316) peptide and its S287A mutant (corresponding to S216A mutation in human CDC25C) were prepared as previously described^8^. The Y283A mutant form of CDC25C peptide (GST-CDC25C (254-316)-Y283A) was produced by using the QuickChange^TM^ site-directed mutagenesis kit from Stratagene (La Jolla, CA). The wildtype GST-CDC25C construct was used as DNA template in a PCR reaction with the following primers: AAACAGAAGCCGCCTTGCTCGCTCACCTTCTATGCC and GGCATAGAAGGT-GAGCGAGCAAGGCGGCTTCTGTTT. The peptides were expressed in the E-coli strain BL21 (DE3)plsS (Strategene, La Jolla, CA) and purified using glutathione agarose beads (Amersham Pharmacia Biotech) as previously reported^8^.

**Standard Biochemical Assays.** Immunoprecipitations (IP), kinase assays (KA), phospho-amino acid analyses (PAA), and Western blot analysis (WB) using the enhanced chemiluminescence (ECL) detection system (Amersham Pharmacia Biotech) were performed, as described in detail in previous publications^1,2,5,6,8-12^.

**Matrix-assisted laser desorption/ionization–time-of-flight Mass Spectrometry.** The services of Applied Biomics (Hayward, CA, USA) were used for the identification of phosphorylation sites by MALDI-TOF/TOF following a standard protocol. In brief, after the *in vitro* kinase reaction with recombinant murine SYK, phosphorylated recombinant human CDC25C samples were digested in solution overnight at 37^o^C with trypsin. Supel-Tips (Sigma-Aldrich) were used for phosphopeptide enrichment. Tryptic peptides were desalted and concentrated using the Millipore C18 reverse phase Zip-Tips column (ZTC 18S096, Millipore, USA), eluted in 0.5 μL of matrix solution (α-cyano-4-hydroxycinnamic acid [5 mg/mL in 50% acetonitrile, 0.1% trifluoroacetic acid, 25 mmol/L ammonium bicarbonate]), and spotted on the MALDI plate (model ABI 01-192–6-AB). MALDI-TOF MS (matrix-assisted laser desorption/ionization–time-of-flight MS) was performed on an AB Sciex Proteomics Analyzer (AB Sciex, Foster City, CA, USA). MS spectra were acquired in reflection positive ion mode, averaging 4000 laser shots/ per spectrum. A virtual digest was done by submitting protein sequences of interest to University of California–San Francisco Protein Prospector (<http://prospector.ucsf.edu/> prospector/mshome.htm). The MS precursors matching the virtual digest were submitted for collision induced dissociation (CID) fragmentation. Peptide masses and associated CID spectra were submitted to GPS Explorer workstation equipped with MASCOT search engine (Matrix Science) to search the database of Swiss-Prot. Candidates with either a protein score confidence interval percentage or ion confidence interval percentage of >95% were considered significant. The spectra of all peptides containing potential phosphorylation sites were manually evaluated for the loss of phosphate.

**Gene Set Enrichment Analysis (GSEA)**.^13^ Rank ordered T-values for SYK induced samples were processed for enrichment of Biological Process Gene Ontology terms using a supervised approach implemented in GSEA2.08 (Broad institute). SYK-induced genes were rank-ordered according to the T-value (positive T-values indicated an increase in expression in SYK induced cells). These ranked ordered genes were screened for enrichment in “Biological Process” Gene Ontology terms for 12625 transcripts representing 9096 genes using weighted Kolmogorov-Smirnov statistics implemented in GSEA (GSEA v2.08 (Broad Institute). GSEA evaluated significance of the over-representation of Gene Ontology terms correlated or anti-correlated with SYK induction by calculating the Enrichment Score (ES) that represents the difference between the observed rankings from the expected null assuming a random rank distribution utilizing an empirical permutation test procedure that randomly assigned gene names to the rank ordered T-values (“GSEA Preranked” algorithm). Nominal P-values were computed by comparing the tails of the ES scores for observed and permutation-generated null distributions following 10,000 permutations. Gene sets containing less than 15 genes or more than 500 genes were filtered for analysis yielding 475 terms (compiled from the file, “gseaftp.broadinstitute.org://pub/gsea/gene_sets/c5.bp.v3.0.symbols.gmt”, Broad Institute) of which 136 gene sets were down regulated in SYK induced cells and 32 gene sets were down regulated with nominal P-values less than 0.05. Previously published global gene expression data (GSE11436) in thymocytes harvested from wildtype and ATM^-/-^ mice were compared to identify ionizing radiation (IR) -responsive genes that are ATM-dependent. Rank ordered T-values for SYK induced samples were processed for enrichment of downstream targets of ATM in irradiated mice. ATM-dependent, G_2_/M checkpoint activated gene expression changes in irradiated mouse cells were examined for enrichment of up- or down-regulated genes in SYK-induced human U373 cells. In this GSEA analysis, 861 down-regulated probe sets on the mouse gene chip were mapped to 388 human ortholog genes on the Affymetrix U95Av2 gene chip and 454 up regulated mouse probe sets were mapped to 235 human genes. Rank-ordered fold difference values from the comparison of irradiated wildtype and ATM^-/-^ mouse thymocytes and rank- ordered T-values for SYK-induced samples were processed for enrichment of downstream targets of human ATM. Human ATM target genes were identified from gene expression studies whereby genes were either down- or up-regulated in irradiated wildtype vs. ATM-mutant lymphoblasts^14^. Forty human radiation-responsive ATM target genes were down-regulated in wildtype lymphoblasts (irradiated/control ratios) and 22 genes exhibited increased expression in wildtype compared to ATM-mutant cells. These 2 groups of human ATM target genes were examined for enrichment of the human orthologs of radiation-responsive mouse ATM target genes as well as for SYK target genes. GSEA normalized the ES score for each gene relative to the set sizes yielding a normalized enrichment score (NES) to enable calculation of a false discovery rate (FDR) corresponding to each NES.

**References**

1. Uckun, F.M., Qazi, S., Ma, H., Tuel-Ahlgren, L., Ozer, Z. STAT3 is a substrate of SYK tyrosine kinase in B-lineage leukemia/lymphoma cells exposed to oxidative stress. *Proc. Natl. Acad. Sci. USA* **107**: 2902-7 (2010)

2. Uckun, F.M., Ma, H., Zhang, J., Ozer, Z., Dovat, S., Mao, C., Ishkhanian, R., Goodman, P., Qazi, S. Serine phosphorylation by SYK is critical for nuclear localization and transcription factor function of Ikaros. Proc Natl Acad Sci U S A. 109:18072-7 (2012)

3. Uckun, F.M., Stork, L., Seibel, N., Sarquis, M., Bedros, C., Sather, H., Sensel, M., Reaman, G.H., Gaynon, P.S. Residual bone marrow leukemic progenitor cell burden after induction chemotherapy in pediatric patients with acute lymphoblastic leukemia. *Clin Cancer Res.* 6(8): 3123-3130 (2000).

4. Rowley, R.B., Bolen, J.B., Fargnoli. J. Molecular cloning of rodent p72syk. *J. Biol. Chem*. 270: 12659-12664 (1995)

5. Uckun, F.M., Ozer, Z., Qazi, S., Tuel-Ahlgren, L., Mao, C. Polo-like kinase 1 (PLK1) as a molecular target to overcome Syk-mediated resistance of B-lineage acute lymphoblastic leukemia cells to oxidative stress. *British Journal of Haematology* **148**: 714-25 (2010)

6. Uckun, F.M., Tuel-Ahlgren, L., Waddick, K.G., Jun, X., Jin, J., Myers, D.E., Rowley, R.B., Burkhardt, A.L., Bolen, J.B. Physical and functional interactions between Lyn and p34cdc2 kinases in irradiated human B-cell precursors*. J. Biol. Chem*. 271, 6396-6397 (1996)

7. [Uckun FM](http://www.ncbi.nlm.nih.gov/pubmed?term=Uckun FM%5BAuthor%5D&cauthor=true&cauthor_uid=10741549), [Mao C](http://www.ncbi.nlm.nih.gov/pubmed?term=Mao C%5BAuthor%5D&cauthor=true&cauthor_uid=10741549), [Vassilev AO](http://www.ncbi.nlm.nih.gov/pubmed?term=Vassilev AO%5BAuthor%5D&cauthor=true&cauthor_uid=10741549), [Huang H](http://www.ncbi.nlm.nih.gov/pubmed?term=Huang H%5BAuthor%5D&cauthor=true&cauthor_uid=10741549), [Jan ST](http://www.ncbi.nlm.nih.gov/pubmed?term=Jan ST%5BAuthor%5D&cauthor=true&cauthor_uid=10741549). Structure-based design of a novel synthetic spiroketal pyran as a pharmacophore for the marine natural product spongistatin 1. [Bioorg Med Chem Lett.](http://www.ncbi.nlm.nih.gov/pubmed/10741549) 10(6):541-5 (2000)

8. Kumogai, A., Guo, A., Emami, K.H., Wang, S., Dunphy, W.G. The Xenopus Chk1 protein kinase mediates a caffeine-sensitive pathway of checkpoint control in cell-free extracts. J Cell Biol 142, 1559-1569 (1998)

9. Mahajan, S., Vassilev, A., Sun, L., Ozer, Z., Mao, C., Uckun, F.M. Transcription factor STAT5A is a substrate of Bruton's tyrosine kinase in B cells. *J Biol Chem****.* 276**: 31216-31228 (2001)

10. Uckun, F.M., Burkhardt, A.L., Jarvis, L., Jun, X., Stealey, B., Dibirdik, I., Myers, D.E., Tuel-Ahlgren, L., Bolen, J.B. Signal transduction through the CD19 receptor during discrete

developmental stages of human B-cell ontogeny. *J Biol Chem.* **268**: 21172-21184 (1993)

11. Uckun, F.M., Waddick, K.G., Mahajan, S., Jun, X., Takata, M., Bolen, J., Kurosaki, T0. BTK as a mediator of radiation-induced apoptosis in DT40 lymphoma B-cells. Science **273**, 1096-1100 (1996)

12. Uckun, F.M, Goodman, P., Ma, H., Dibirdik, I., Qazi, S. CD22 Exon 12 Deletion as a Novel Pathogenic Mechanism of Human B-Precursor Leukemia. *Proc. Natl. Acad. Sci. USA* **107**:16852-16857 (2010)

12. Chen P, Luo C, Deng Y, Ryan K, Register J, Margosiak S, Tempczyk-Russell A, Nguyen B, Myers P, Lundgren K, Kan CC, O'Connor PM. (2000) The 1.7 Å crystal structure of human cell cycle checkpoint kinase Chk1: implications for Chk1 regulation. *Cell* 100(6):681-92

13. Subramanian A, Tamayo P, Mootha VK, Mukherjee S, Ebert BL, Gillette MA, Paulovich A, Pomeroy SL, Golub TR, Lander ES, Mesirov JP. (2005) Gene set enrichment analysis: A knowledge-based approach for interpreting genome-wide expression profiles. PNAS 102: 15545-15550

14. Innes CL, Heinloth AN, Flores KG, Sieber SO, Deming PB, Bushel PR, Kaufmann WK, Paules RS. (2006) ATM requirement in gene expression responses to ionizing radiation in human lymphoblasts and fibroblasts. Mol Cancer Res 4: 197-207

**FIGURES AND FIGURE LEGENDS**


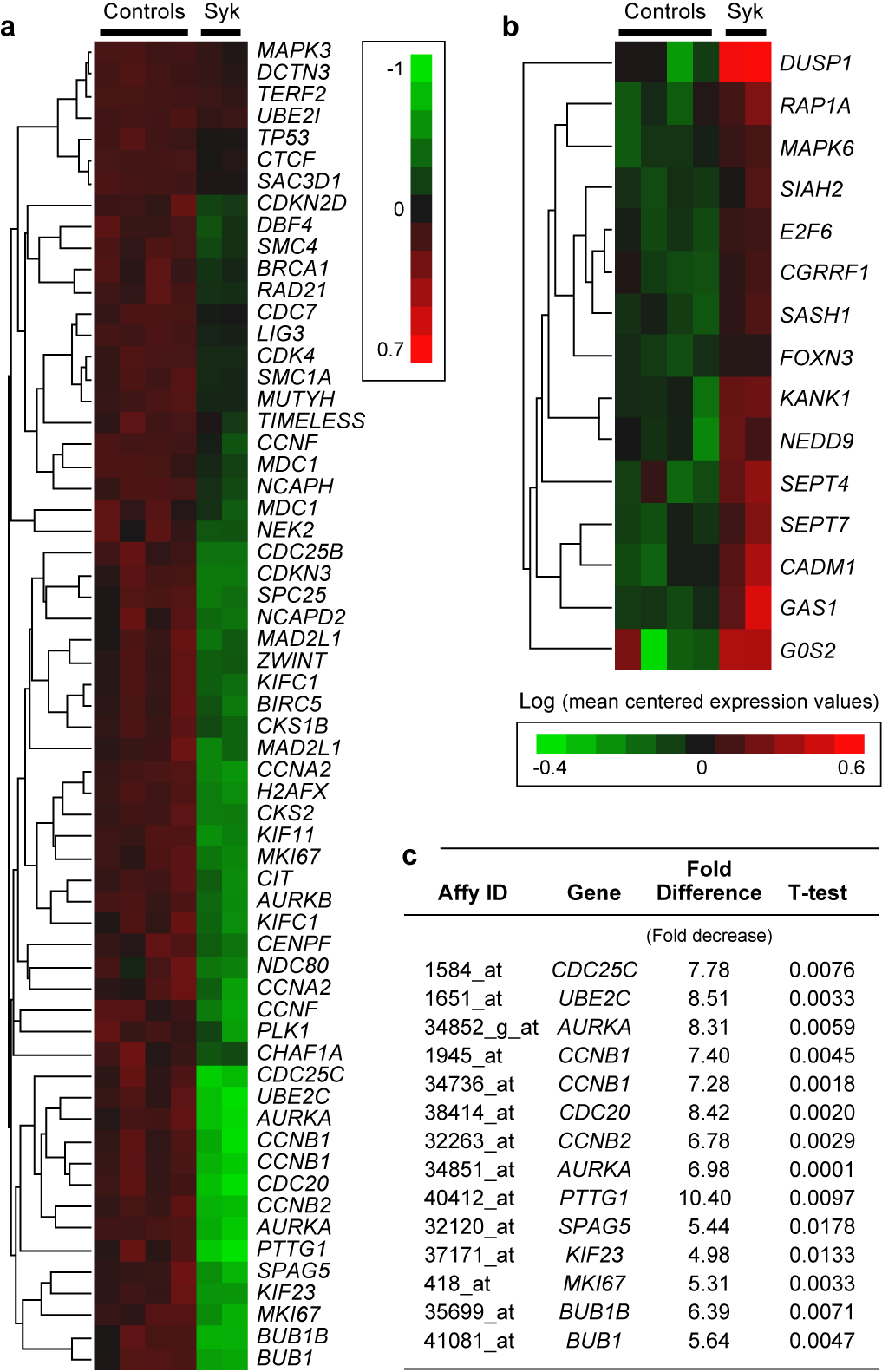


**Figure S1.** **Perturbation of Cell Cycle Transcriptome in an ecdysone-inducible mammalian expression system for SYK.**  Pon-A (10 µM)-induced SYK induction leads to upregulation of SYK-responsive genes. Induction of SYK expression with Pon-A (10 µM) caused a pronounced perturbation of the transcriptome with altered expression of multiple evolutionarily conserved mitotic checkpoint genes. The heat map represents the color-coded log-transformed mean centered (relative to controls) expression values. A one-way hierarchical clustering technique was used to organize the expression patterns such that genes having similar expression on days 1 and 2 after SYK induction were grouped together (JMP Software, SAS, Cary, NC). The cluster figure depicts log_10_ transformed expression values mean centered to the 4 control samples (green shows a decrease and red an increase of gene expression levels in SYK-induced cells). Raw data were deposited (Gene Expression Omnibus Series GSE18798). [a] Cell cycle related genes (53 genes represented by 61 probe sets) significantly down regulated after induction of SYK.  [b] Cell cycle related genes (15 genes represented by 15 probe sets) that were significantly upregulated after induction of SYK.  [c] List of most significantly affected genes exhibiting the greatest fold difference values.


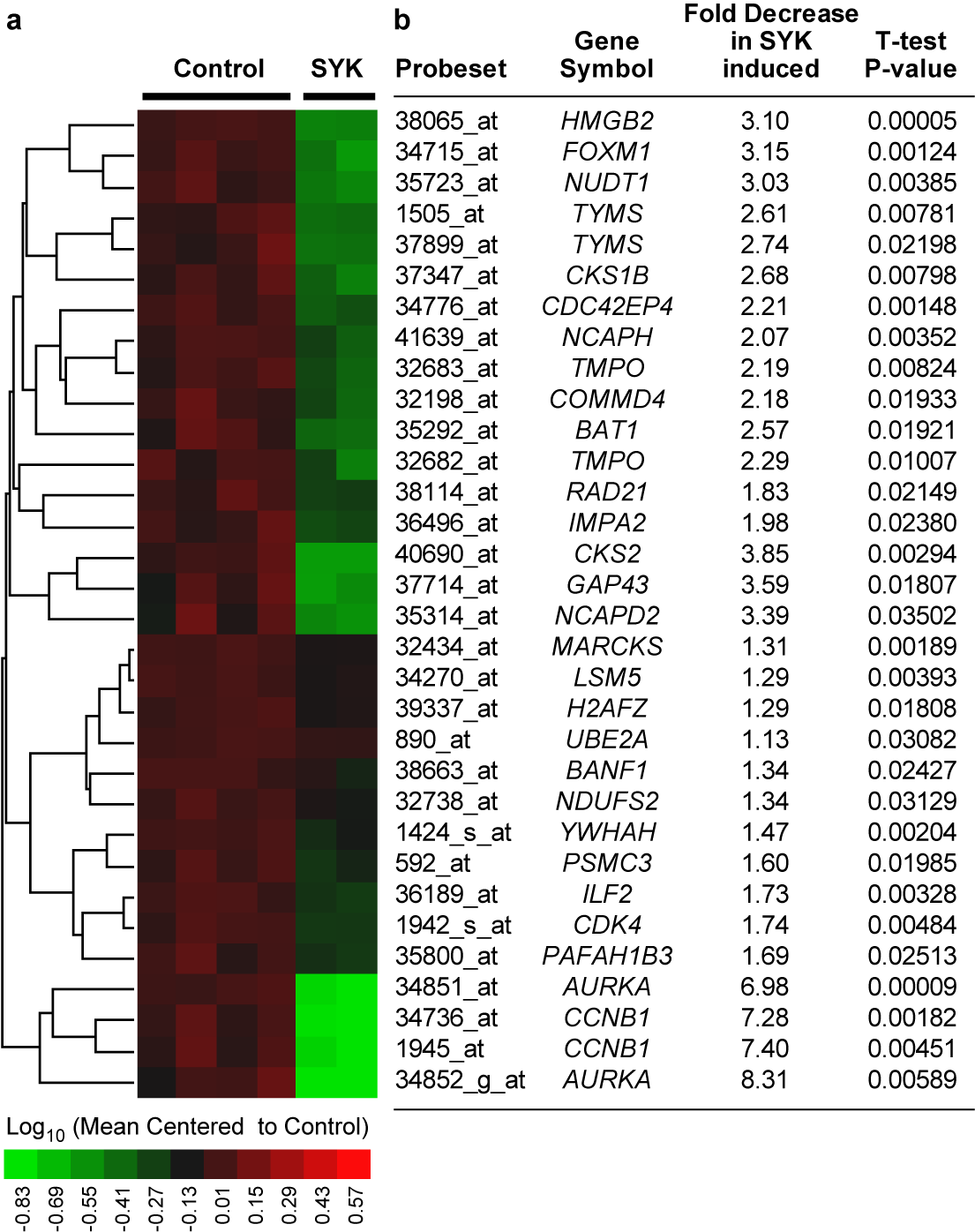


**Figure S2. SYK induction causes down-regulation of ATM-dependent cell cycle genes.** [a] Previously published global gene expression data (GSE11436) in thymocytes harvested from wildtype and ATM^-/-^ mice were compared to identify ionizing radiation (IR) -responsive genes that are ATM-dependent. We compared WT versus ATM^-/-^ cells for radiation (5 Gy)-induced gene expression changes using Student’s T-tests (2-sample, P-value cut off < 0.05). Duplicate samples for each treatment were hybridized to Affymetrix Mouse Genome 430A 2.0 gene chips and the scaled signal expression values were log_10_ transformed for statistical comparisons. The heat map represents the color-coded log-transformed mean centered (relative to controls) expression values. [b] Signal expression values were cross-referenced to their human homologues (http://www.affymetrix.com/analysis/index.affx) that were down- regulated following SYK induction in the U373 expression system.


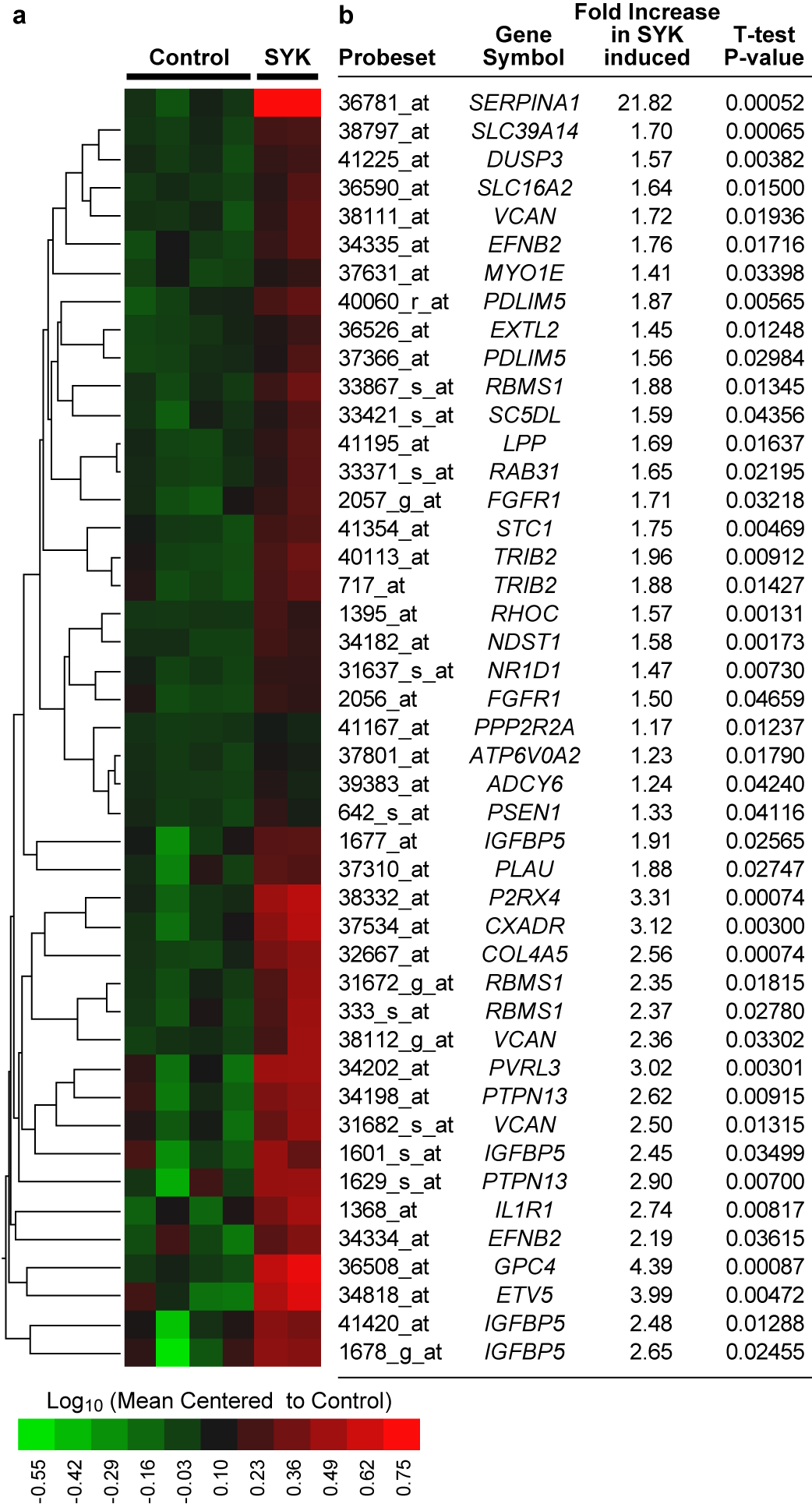


**FIG S3. SYK Induction Causes Upregulation of ATM-dependent G_2_/M Checkpoint Gene Cassette.** Previously published global gene expression data (GSE11436) in thymocytes harvested from wildtype and ATM^-/-^ mice were compared to identify ionizing radiation (IR) -responsive genes that are ATM-dependent. We compared WT versus ATM^-/-^ cells for radiation (5 Gy)-induced gene expression changes using Student’s T-tests (2-sample, P-value cut off < 0.05). Duplicate samples for each treatment were hybridized to Affymetrix Mouse Genome 430A 2.0 gene chips and the scaled signal expression values were log_10_ transformed for statistical comparisons. Signal expression values were cross-referenced to their human homologues (http://www.affymetrix.com/analysis/index.affx) that were up-regulated following SYK induction in the U373 expression system. Depicted are SYK-induced expression changes of the ATM-dependent genes that are expressed at significantly lower levels in irradiated ATM^-/-^ cells than in irradiated wildtype cells. *SERPINA1* showed 21.82 fold increase and a cluster of 12 genes (*COL4A5, CXADR, EFNB2, ETV5, GPC4, IGFBP5, IL1R1, P2RX4, PTPN13, PVRL3, RBMS1, VCAN*) with greater than 2-fold increase following SYK induction. The heat map represents the color-coded log-transformed mean centered (relative to controls) expression values. A one-way hierarchical clustering technique was used to organize the expression patterns such that genes having similar expression on days 1 and 2 after SYK induction were grouped together (JMP Software, SAS, Cary, NC). The cluster figure depicts log_10_ transformed expression values mean centered to the 4 control samples (green shows a decrease and red an increase of gene expression levels in *SYK*-induced cells). Raw data were deposited (Gene Expression Omnibus Series GSE18798).
